# Structural basis for small molecule targeting of the programmed death ligand 1 (PD-L1)

**DOI:** 10.18632/oncotarget.8730

**Published:** 2016-04-13

**Authors:** Krzysztof M. Zak, Przemyslaw Grudnik, Katarzyna Guzik, Bartosz J. Zieba, Bogdan Musielak, Alexander Dömling, Grzegorz Dubin, Tad A. Holak

**Affiliations:** ^1^ Malopolska Centre of Biotechnology, Jagiellonian University, Gronostajowa, Krakow, Poland; ^2^ Faculty of Biochemistry, Biophysics and Biotechnology, Jagiellonian University, Gronostajowa, Krakow, Poland; ^3^ Department of Organic Chemistry, Faculty of Chemistry, Jagiellonian University, Ingardena, Krakow, Poland; ^4^ Department for Drug Design, University of Groningen, A. Deusinglaan, Groningen, The Netherlands; ^5^ Max Planck Institute for Biochemistry, Am Klopferspitz, Martinsried, Germany

**Keywords:** immunotherapy, checkpoint inhibitor, small molecule, X-ray structure

## Abstract

Targeting the PD-1/PD-L1 immunologic checkpoint with monoclonal antibodies has provided unprecedented results in cancer treatment in the recent years. Development of chemical inhibitors for this pathway lags the antibody development because of insufficient structural information. The first nonpeptidic chemical inhibitors that target the PD-1/PD-L1 interaction have only been recently disclosed by Bristol-Myers Squibb. Here, we show that these small-molecule compounds bind directly to PD-L1 and that they potently block PD-1 binding. Structural studies reveal a dimeric protein complex with a single small molecule which stabilizes the dimer thus occluding the PD-1 interaction surface of PD-L1s. The small-molecule interaction “hot spots” on PD-L1 surfaces suggest approaches for the PD-1/PD-L1 antagonist drug discovery.

## INTRODUCTION

Cancer cells avoid immune surveillance by overexpressing negative immunologic regulators. In normal conditions these regulators provide checkpoints that restrain the immune system against targeting self-antigens. However, when overproduced by cancer cells, they protect those cells against hosts' immune system. Even more, such interactions result in the exhaustion (loss of function) of the antigen-specific effector T-cells. The immunosuppressive environment created in these ways permits the cancer cells to proliferate unrestrained. The reversal of these effects by blocking the interaction of the negative immunologic regulators on cancer cells, or by blocking their receptors on immune effector cells, should in principle help to eliminate cancer [recent reviews: 1–5]. Indeed, the proof-of-concept was established with the hope-rising results of clinical trials evaluating ipilimumab, an antibody blocking the immune checkpoint receptor CTLA-4, resulting in the FDA acceptance for the combined anti-cancer treatment in 2011 [3]. Even more encouraging results were more recently obtained for nivolumab and pembrolizumab, antibodies targeting the programmed death 1 (PD-1)/PD-1 ligand (PD-L1) immune checkpoint. The tumor responses in clinical trials in melanoma were impressive enough to merit the accelerated approval of these antibodies in 2014 [2] [6–7] [http://www.fda.gov/NewsEvents/­Newsroom/PressAnnouncements/ucm436534.htm]. Moreover, in 2015, after demonstrating unprecedented results in clinical trial in the metastatic squamous NSCLC, nivolumab has gained FDA acceptance in this indication, becoming the first monotherapy in more than 15 years to demonstrate proven superior overall survival compared to the standard of care [http://www.fda.gov/Drugs/InformationOnDrugs/ApprovedDrugs/ucm436566.htm]. Numerous further clinical trials are currently in progress [8].

Inhibition of the PD-1/PD-L1 axis is also feasible by targeting PD-L1. Objective responses were observed in melanoma, NSCLC and certain other solid tumors in the phase I clinical trial evaluating monoclonal antibody BMS-936559 (MDX1105) [9]. Similar encouraging results were obtained with MEDI4736 antibody in NSCLC patients [10]. The third anti-PD-L1 antibody currently evaluated in clinics, MPDL3280A, demonstrated positive results in melanoma, NSCLC and genitourinary cancers [11]. It was also granted an FDA breakthrough designation for the metastatic urothelial bladder cancer after demonstrating impressive results in the phase I trial in which tumor shrinkage was observed in 43% of patients [12]. Additional phase I and phase II trials of anti-PD-L1 antibodies are currently in progress [8].

Ongoing trials evaluating the PD-1 and PD-L1 targeting antibodies in multiple indications in cancer portend further rapid development. Moreover, initial results demonstrate association between PD-L1 status and the response to the treatment [13]. A reliable biomarker would likely result in further improvement in observed response rates by rational patient selection. Overall, it is currently expected that antibodies targeting immunologic regulators will soon become a significant aspect of the therapy within a variety of malignancies [8].

This impressive clinical development of the antibodies that interfere with the PD-1/PD-L1 axis is in contrast to the development of small-molecule modulators for this interaction. Several low-molecular weight immunomodulators targeting the PD-1/PD-L1 signaling pathway were reported. These are based on peptidomimetics [14–15] and macrocyclic peptides [16]. More recently, in 2015, the 1,2,4-oxadiazole- and 1,2,4-thiadiazole-based inhibitors that contain an extensive peptidic component have been proposed to suppress and/or inhibit the PD-1/PD-L1 signaling pathway, although no data supporting that action has been provided [17]. We have tested several of the described peptidomimetic agents and could show no direct binding to the target PD proteins, suggesting that possibly other targets are involved. Bristol-Myers Squibb has recently disclosed the first entirely nonpeptidic molecules which are claimed to be “useful as inhibitors of the PD-1/PD-L1 protein/protein interaction”, although no detailed information was provided [18].

Clearly, progress of small molecule modulators of PD-1/PD-L1 pathway is lagging behind that of antibodies, which is partially related to insufficient structural information to guide rational design and development. The structure of the complex of the murine PD-1 and human PD-L1 revealed the overall binding mode for the PD-1/PD-L1 interaction [19], but relatively low sequence identity of human and murine orthologues limited the relevance of this finding for drug design. The structures of human PD-1 (PDB 3RRQ) and human PD-L1 (PDB 3BIS, 3FN3, 4Z18, 5C3T) have been determined, but those in turn did not account for significant plasticity within the human PD-1 upon complex formation demonstrated only very recently by our structure of the fully human PD-1/PD-L1 complex [20]. Although the above structures provided a complete description of the interaction, the flat surface of the protein-protein interface still complicates drug design efforts in the absence of structural information on the small-molecule inhibitors in complex with either PD-1 or PD-L1 to guide further rational drug development.

Herein, we report characterization of the interaction of Bristol-Myers Squibb (BMS) compounds with the target protein. We show that they act by directly binding to PD-L1 and not PD-1 and effectively dissociate a preformed PD-1/PD-L1 complex *in vitro*. We further show that they inhibit the PD-1/PD-L1 interaction by inducing PD-L1 dimerization through PD-1 interacting surface. We provide the first crystal structures of a small-molecule inhibitors bound to its target PD protein, in this case the PD-L1 dimer. Together with our biochemical data, the provided insight into this protein-inhibitor interaction and detailed definition of the binding site “hot spots” should facilitate more dynamic progress in the development of the PD-1/PD-L1 immune checkpoint modulators.

## RESULTS

### BMS-8 and BMS-202 bind to PD-L1 and dissociate the human PD-1/PD-L1 complex in NMR assays

**Figure 1:**
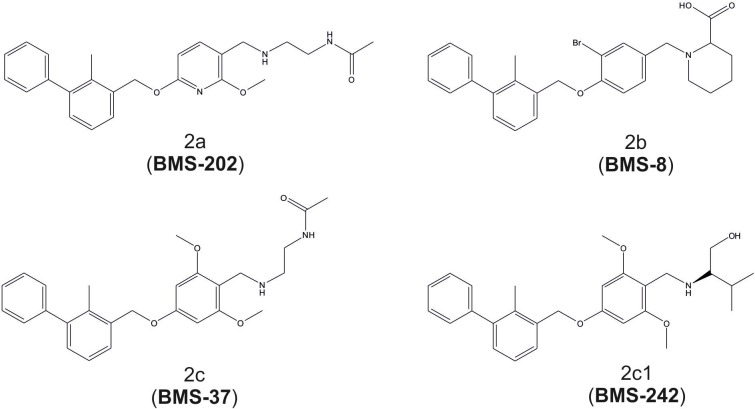
Chemical structures of the BMS-8, -37, -202 and -242 inhibitors of PD-1/PD-L1 interaction Compound numbering according to patent application WO2015034820 (A1).

The small-molecule PD-1/PD-L1 inhibitors of Bristol-Myers Squibb are based on the (2-methyl-3-biphenylyl)methanol scaffold [18]. In the original disclosure, the inhibition of the formation of PD-L1/PD-1 protein/protein complex was demonstrated in a homogenous time-resolved fluorescence (HTRF) assay, but mechanistic or structural features, including the actual target protein, were not disclosed. We have synthesized and tested four examples of their compounds with the reported inhibition IC_50_ towards the PD-L1/PD-1 complex in the range of 18 to 200 nM, the lowest IC_50_ values in the series of about 200 compounds. The compounds that we have studied are 8, 37, 202 and 242 in the patent, and these are designated herein as BMS-8, BMS-37, BMS-202 and BMS-242, respectively (Figure 1).

To unambiguously validate if these compounds bind to PD-1 or PD-L1, we used NMR, a method that monitors the direct binding of a small molecule to its target protein [21–22]. We performed binary titrations, using the “SAR-by-NMR” approach [21]. The method relies on monitoring chemical shift perturbations in 2D ^1^H-^15^N HMQC spectra of ^15^N-labeled proteins upon interaction with tested ligands. The ^15^N labeled PD-L1 was titrated with increasing amount of tested compound while the ^1^H-^15^N signals were monitored using 2D HMQC NMR experiment. Significant shifts in the correlation NMR signals of PD-L1 upon addition of each tested compound documented their direct binding to PD-L1 (Figure S1 and S2). No significant shifts were observed upon titration of the ^15^N labeled PD-1 with the tested compounds, demonstrating their specificity for binding to PD-L1 (data not shown).

The capability of BMS-202 to block the PD-1/PD-L1 interaction was evaluated in the NMR based AIDA assay. The assay monitors broadening of resonance signals upon protein-protein complex formation related to increased relaxation time of the higher molecular weight entities [23–25]. To this end the ^15^N labeled PD-1 was first titrated with the non-labeled PD-L1 until no further changes in the linewidth of the ^1^H-^15^N resonance peaks was observed as monitored by HMQC (Figure S3). In such conditions the sample contained slight excess of PD-L1 over PD-1. The molecules formed a tight complex which molecular weight was estimated at around 30 kDa based on the relaxation time analysis. This corresponds well with the calculated mass of a 1:1 complex (27.5 kDa). Upon titration of this preformed complex with BMS-202 narrowing of ^1^H-^15^N signals was observed which provides direct evidence of the complex dissociation.

### BMS-8 and BMS-202 selectively induce thermal stabilization of PD-L1

To facilitate the choice of the compound suitable for structural studies, we evaluated the relative affinity of BMS-8 and BMS-202 towards PD-L1 and PD-L2 using differential scanning fluorimetry (DSF) [26]. DSF monitors thermal unfolding of the protein in the presence of a fluorescent dye sensitive to the changes in hydrophobicity (protein denaturation). Binding of small-molecule ligands induces thermal stabilization of the target protein which is proportional to compound affinity [27]. PD-L1 exhibited a relatively low melting temperature (T_m_) of 35.4°C (Figure S4A). BMS-8 stabilized the thermally induced unfolding of PD-L1 by 9.4°C (T_m_=44.8°C), whereas BMS-202 by 13°C (T_m_=48.4°C). PD-L2 was characterized by a comparable melting temperature as PD-L1 (38.2°C), however, neither BMS-8 nor BMS-202 significantly affected the T_m_ value (38.6°C and 35.6°C, for BMS-8 and BMS-202, respectively) (Figure S4B). These results further confirm the interaction of both tested compounds with hPD-L1 and indicate that BMS-8 exhibits lower affinity compared to BMS-202. Furthermore, the presented data suggests that both compounds bind specifically only to PD-L1, but not PD-L2.

### Structural basis of the interaction of BMS-202 with PD-L1

**Figure 2:**
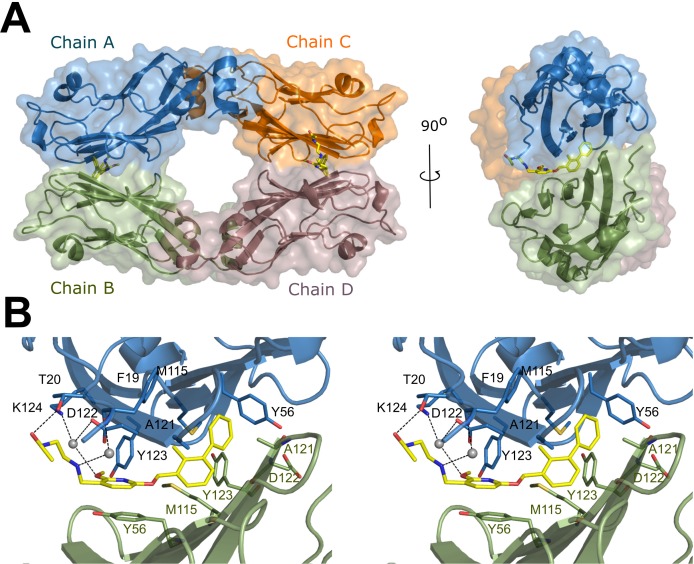
Crystal structure of BMS-202/PD-L1 complex **A.** Within the asymmetric unit four molecules of PD-L1 (mixed ribbon/surface representation) are organized into two dimers (green and blue, and orange and brown). Each dimer binds a single molecule of BMS-202 (yellow) at the dimer interface. **B.** Detailed interactions of BMS-202 at the binding cleft of PD-L1 dimer (stereoview). BMS-202 binds at a hydrophobic cavity formed upon PD-L1 dimerization. Color coding as in panel a. Water molecules are represented by grey spheres. Hydrogen bonds are shown as black dotted lines.

Having confirmed the affinity of BMS-202 towards PD-L1 and its ability to dissociate the PD-1/PD-L1 interaction, we crystallized the compound in the complex with the target protein. The obtained crystals diffracted to the 2.2 Å resolution (Table 1). Four protein molecules found in the asymmetric unit are organized into two dimers with one inhibitor molecule located at the interface of each dimer (i.e. the stoichiometry of the BMS-202 : PD-L1 in the complex is 1: 2, respectively) (Figure 2A). No additional inhibitor molecules were found within the structure. The protein dimer exhibits a pseudo 2-fold rotational symmetry around an axis parallel to the long axis of the PD-L1 molecule. The inhibitor is located roughly perpendicular to the dimer pseudo-symmetry axis and its disposition does not follow the symmetry of the dimer (Figure 2A). Both the binding site of the inhibitor and the intermolecular interactions within the dimer involve the PD-1 interaction surface of PD-L1, providing a rationale of the mechanism of action of BMS-202 (ie. dimerization related occlusion of PD-1 interaction surface).

**Table 1:** Data Collection and Refinement Statistics (Molecular Replacement)

	PD-L1 in complex with the small-molecule inhibitor (BMS-202)	PD-L1 in complex with the small-molecule inhibitor (BMS-8)
**Data collection**		
Space group	P 21 21 21	P 2 21 21
Cell dimensions		
*a*, *b*, *c* (Å)	40.83, 85.09, 161.77	34.31, 55.18, 141.77
α, β, γ (°)	90, 90, 90	90, 90, 90
Resolution (Å)	42.55 - 2.2 (2.279 - 2.2)*	47.26 - 2.3 (2.38 - 2.3)*
*R*_merge_	0.05 (0.272)*	0.093 (0.465)*
*I* / σ*I*	23 (6.3)*	12.4 (3.6)*
Completeness (%)	99.3 (99.0)*	99.9 (100.0)*
Redundancy	3.4 (3.5)*	6.8 (7.4)*
**Refinement**		
Resolution (Å)	2.2	2.3
No. reflections	29405 (2879)	12626 (1191)
*R*_work_ / *R*_free_	0.2064 / 0.2582 (0.2666/0.3452)	0.2390 / 0.2970 (0.2691/0.3845)
No. atoms	4122	1968
Protein	3848	1906
Ligand/ion	66	32
Water	208	30
*B*-factors	48.27	45.15
Protein	48.61	45.17
Ligand/ion	47.08	42.77
Water	42.31	46.53
R.m.s. deviations		
Bond lengths (Å)	0.009	0.014
Bond angles (°)	1.005	1.85

* Values in parentheses are for highest-resolution shell.

**Figure 3:**
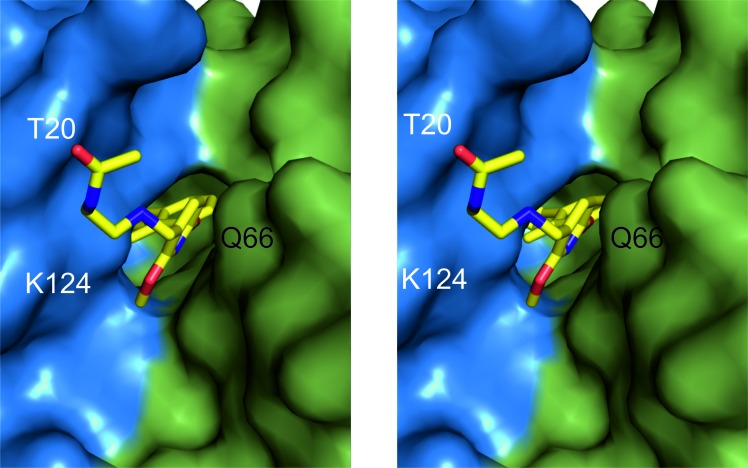
BMS-202 binds inside a cylindrical cleft at PD-L1 dimer interface Hydrophobic pocket accommodating the inhibitor and formed at PD-L1 dimer interface is shown from the solvent accessible side (stereoview). *N*-(2-aminoethyl)acetamide moiety of the inhibitor is visible. The PD-L1 molecules forming the dimer are colored blue and green for chain A and B, respectively.

Both inhibitor molecules are well defined by the electron density allowing unambiguous positioning of all moieties (Figure S5). The inhibitor inserts deep into a cylindrical, hydrophobic pocket created at the interface of two monomers within the dimer (Figure 3 and Figure S6). The pocket is open to the solvent on one side of the dimer and restricted by the sidechain of _A_Tyr56 on the opposite side (the protein molecules are annotated by subscripts A, B, C and D according to the chain arrangement in the crystal structure; the inhibitor disposition is described here based on AB dimer and is similar in CD dimer unless noted otherwise). The 2-methylbiphenyl core of the inhibitor anchors at the very bottom of the pocket (Figure 2B and Figure S7). The distal phenyl ring within the biphenyl creates a T-stacking interaction with the sidechain of _A_Tyr56 and is further stabilized by π-alkyl interactions with the sidechains of _A_Met115 and _B_Ala121. The orientation of central methyl-phenyl ring is roughly related to that of the distal ring by a pseudo glide plane through ring connecting bond and roughly 45° to the ring planes. The major interactions of the central ring involve the hydrophobic interactions with _A_Ala121 and _B_Met115. The methyl of this ring provides additional interactions in a pocket formed by _A_Met115, _A_Ala121 and _A_Tyr123 (Figure 2B and Figure S7). Moreover the methyl group serves to turn the two phenyl groups out of coplanarity to preorganize the ligand binding conformation. The methoxy-pyridine moiety provides a significant contribution to the compound binding including π-π stacking with the ring of _B_Tyr56 and a number of polar interactions with the A monomer including the carbonyl-π interaction with the backbone of _A_Ala121, the anion-π interaction with the sidechain of _A_Asp122, a water-mediated lone-pair-π interaction with the backbone of _A_Phe19 and the water-mediated interactions of the methoxyl group with the sidechains of _A_Asp122 and _A_Lys124 and the backbone carbonyl of _A_Tyr123. The extended *N*-(2-aminoethyl)acetamide moiety of the inhibitor provides additional electrostatic interactions, but only with the A monomer (and corresponding C monomer for the second inhibitor molecule contained in the asymmetric unit). A water-mediated interaction with the backbone carbonyl of _A_Phe19 is observed for both inhibitor molecules contained in the asymmetric unit, but further interactions of the distal parts of the acetamide group differ between the monomers. This is unrelated to crystal packing since no symmetry related molecules locate near the described moiety, but rather seems an effect of its flexibility (evidenced by high temperature factors, Figure S8C) and a solvent-exposed character of the binding site in this region (the described inhibitor moiety interacts with surface residues rather than within a defined pocket; Figure 2B, Figure 3). In one of the dimers (AB) contained in the asymmetric unit the acetamide moiety contributes a hydrogen bond to the sidechain of _A_Lys124 whereas in the second dimer (CD) with that of _C_Thr20. Overall, the inhibitor-protein interaction is best described as bimodal, spatially divided into hydrophobic and electrostatic parts following the inhibitor bimodal design.

### Crystal structure of PD-L1/BMS-8 complex supports compound induced dimerization of PD-L1

The crystal structure of PD-L1/BMS-202 complex suggests that BMS-202 induces dimerization of PD-L1. To assess if the observed dimerization is induced by crystallization conditions or rather is specific for this class of compounds we crystallized BMS-8 in complex with PD-L1 (Table 1). The crystal structure was solved at 2.3 Å resolution and clear and continuous electron density allowed unambiguous positioning of the inhibitor and all the interacting residues. The asymmetric unit contains two molecules of PD-L1 organized in a dimer identical to that observed in the structure of PD-L1/BMS-202. A single molecule of BMS-8 binds at the interface of the dimer in a pocket almost identical to that found in PD-L1/BMS-202 complex (Figure S8B). The disposition of the buried part of BMS-8 is identical to that observed for BMS-202 and the only differences are observed in the part directed towards the solvent (Figure S8A). Overall, the binding mode of the compound and geometry of PD-L1 dimer are identical in the structures containing BMS-8 and BMS-202 save for detailed interactions of the solvent directed moieties. The fact that crystals of PD-L1/BMS-8 complex were obtained in different conditions and belong to a different space group than those of PD-L1/BMS-202 strongly advocates that PD-L1 dimerization reflects a specific mechanism of action of BMS compounds rather than a crystallization artifact.

### BMS-202 and BMS-8 induce dimerization of PD-L1 in solution

To further assess if BMS compounds induced dimerization of PD-L1 is an effect of tight packing within the crystal or rather constitutes the actual mechanism of action, we checked whether BMS-202 and BMS-8 induce the PD-L1 dimerization in solution. To this end PD-L1 was characterized by size exclusion chromatography in the presence and absence of tested compounds. Apo-PD-L1 yielded a single peak corresponding to a protein of molecular weight of 17 kDa, suggesting a monomeric state of the apoprotein in solution. In the presence of each compound the peak shifted to shorter retention time corresponding to a protein of around 34 kDa, consistent with a calculated molecular weight of a dimer (Figure S9). This indicates the PD-L1 dimerization by both BMS-202 and BMS-8 in solution.

To further evaluate the presumed BMS compound induced PD-L1 dimerization in solution, we titrated PD-L1 with BMS-202, BMS-8, BMS-37 and BMS-242 while monitoring the ^1^H resonance linewidth by NMR. In all the cases, the well resolved narrow resonance peaks in the aliphatic region of ^1^H NMR spectrum of apo-PD-L1 exhibited significant broadening upon addition of each compound indicating significant increase in the molecular weight of the complex (Figure S1B and S2). The molecular weight of each complex estimated from relaxation time analysis was around 30 kDa, which can only be explained by the compound induced PD-L1 dimerization. Similar resonance linewidth broadening was observed in the ^1^H-^15^N HMQC spectra when the ^15^N-labeled PD-L1 was titrated with BMS-202 (Figure S1A).

Overall, the NMR titration experiments support the results obtained in gel filtration indicating that all tested BMS compounds induce dimerization of PD-L1 in solution and that such preformed dimeric state is reflected by the crystal structures provided in this study.

Multiple hydrophobic and electrostatic interactions stabilize the homodimer (Figure 2B and Figure S8B). The interactions are discussed based on PD-L1/BMS-202 structure, but are similar in PD-L1/BMS-8 structure. The hydroxyl group of _A_Tyr56 creates a 2.6 Å-long hydrogen bond and hydrophobic contacts with _B_Asp122 (graphical representation of the interactions is presented in Figure S10 [28]). _B_Ala121 creates a net of hydrophobic interactions with _A_Tyr56 and _A_Ile54, which at the same time interacts with _B_Gly120 also utilizing hydrophobic interactions. Ser117 in the _A_PD-L1 and _B_PD-L1 molecules participate in the 2.9 Å hydrogen bond and in the hydrophobic contacts, as the Ser117 residues are located parallel to each other. Next, _A_Tyr123 joins with both _B_Met115 and _B_Glu58 using hydrophobic contacts and hydrogen bonds between the carboxyl of _B_Glu58 and hydroxyl of tyrosine. _A_Arg113 builds a dense interaction network consisting of a salt bridge, hydrogen bonding and hydrophobic interactions with _B_Glu58 and _B_Asp61 and hydrophobic contacts with _B_Glu60 and _B_Arg113 as well. _B_Arg113 residue also contributes a salt bridge and hydrogen bonding with _A_Asp61 which creates next salt bridge with _B_Arg125. _B_Tyr123 interacts with _A_Glu58 and _A_Met115 through hydrophobic contacts and hydrogen bonds.

## DISCUSSION

Monoclonal antibodies targeting immunologic checkpoints and especially the PD-1/PD-L1 axis provided spectacular results in cancer therapy in the recent years [recent reviews: 1–4]. Given the large number of ongoing clinical trials this success will likely expand in the near future. Development of small molecule inhibitors of PD-1/PD-L1 checkpoint is currently much behind that of the antibodies, but first tool compounds have already been disclosed. The number of announced commercial programs within this novel segment of immunomodulatory molecules and the detailed structural insight provided within this study promise dynamic progress in the field in the near future.

Several low-molecular weight immunomodulators targeting the PD-1/PD-L1 pathway that are based on peptidomimetics [14–15] and macrocyclic peptides [16] were reported. We have tested several of the described peptidomimetics. The results obtained by us did not indicate binding to either of the PD proteins suggesting that these compounds modulate the PD-1/PD-L1 pathway only indirectly (data not shown).

The first group of true small-molecule inhibitors of PD-1/PD-L1 interaction was described by Bristol-Myers Squibb. The BMS inhibitors are derivatives of (2-methyl-3-biphenylyl)methanol. The capability to block the PD-1/PD-L1 complex formation was demonstrated in the HTRF assay in the original disclosure, but no further confirmatory data or structural information was provided to date. Among their most potent examples, BMS-8 and -202 inhibited the formation of the PD-1/PD-L1 complex with the IC_50_ values of 0.146 and 0.018 μM, respectively. Despite the fact that these compounds have poor drug-like properties and as such are unlikely to become lead structures, they serve well as a proof of principle that targeting the PD-1/PD-L1 interaction surface is feasible not only with antibodies, but also with small molecules. Therefore we attempted their detailed characterization in this study. Most importantly, we wished to characterize the hot spots and key interactions at the surface of the target molecule to guide future rational design of more drug-like inhibitors.

We demonstrated that BMS compounds bind to PD-L1 and not PD-1 and presented direct confirmation by NMR that *in vitro* BMS-8 and BMS-202 are capable of dissociating the PD-1/PD-L1 interaction at stoichiometric concentration, consistent with the estimated K_D_ of the PD-1/PD-L1 complex of 8 μM [29]. Quite unexpectedly, however, the compounds induced dimerization of hPD-L1 in solution.

**Figure 4:**
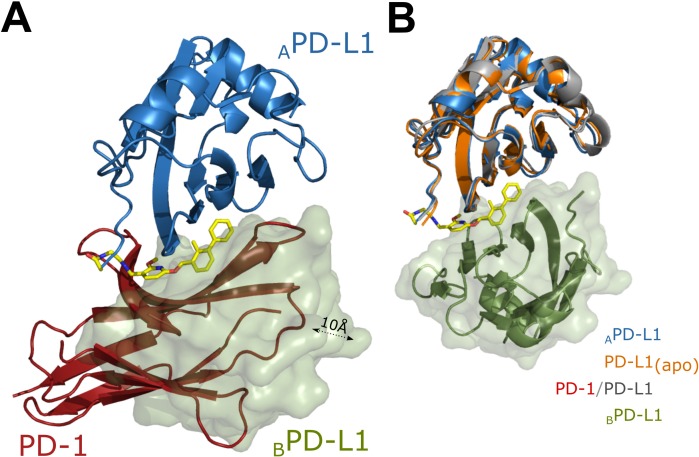
Rationale for inhibition of PD-1/PD-L1 complex formation by BMS-202 (**A**) BMS-202 induced PD-L1 dimer and PD-1/PD-L1 complex were superimposed such that a single molecule of PD-L1 (model A) within the BMS-202 (yellow) induced dimer (blue ribbon- model A, green surface – model B) was superposed with PD-L1 molecule (not shown) within PD-1/PD-L1 complex (PD-1 shown as red ribbon). Model B within PD-L1 dimer and PD-1 do not overlay perfectly (are shifted by around 10Å), but BMS-202 induced dimerization of PD-L1 masks almost the entire PD-1 interaction surface thereby preventing PD-1/PD-L1 interaction. Same is true for BMS-8 containing structure (not shown) (**B**) Superposition of the PD-L1 molecules extracted from apo-PD-L1 (orange ribbon; PDB 5C3T), PD-1/PD-L1 (PDB 4ZQK; PD-L1 shown as grey ribbon; PD-1 is not shown) and PD-L1/BMS-202 complex (model A shown as blue ribbon; BMS-202 shown as yellow sticks) structures demonstrates that PD-L1 does not undergo significant backbone rearrangement upon interaction with BMS-202. Model B of PD-L1/BMS-202 dimer is shown as green ribbon and surface. Same is true for BMS-8 containing structure (not shown).

The presented crystal structures provide explanation for the molecular mechanism of dimerization of PD-L1 and the mechanism of inhibition of PD-L1/PD-1 interaction. The PD-L1 homodimer distantly resembles that of PD-1/PD-L1 (both proteins are characterized by similar IgG like fold [20] in that the two molecules of PD-L1 interact *via* their PD-1 binding surfaces). Nevertheless, overlay of the PD-L1 homodimer with the PD-1/PD-L1 dimer demonstrates that the second PD-L1 molecule within the homodimer does not fully corresponds to the orientation of PD-1 (Figure 4A). In the respective structures, both BMS-202 and BMS-8 are located at the center of the homodimer filling a deep hydrophobic pocket contributing multiple additional interactions between the monomers. The compounds interact with both PD-L1 molecules using hydrophobic surfaces physiologically involved in the PD-1/PD-L1 interaction. This provides the rationale for the activity of BMS compounds in dissociating the PD-1/PD-L1 complex. Not only the inhibitor partially covers the PD-1 binding site in each PD-L1 molecule within the complex, but the interaction of the two monomers fully occludes the PD-1 binding surface thus preventing the interaction with PD-1. Furthermore, neither BMS-202 nor BMS-8 induce changes in the overall protein fold, so that the arrangement of PD-L1 backbone remains the same as in the apo-form and in the PD-1/PD-L1 complex (Figure 4B).

The most important finding of this study is in unambiguous definition of the druggable “hot spots” [30–32] at the surface of PD-L1 suitable for targeting with low-molecular weight inhibitors. Even though the atomic resolution structures of PD-L1 [19] [33] and recently its complex with PD-1 [20] have been published by others and our group providing directions for rational inhibitor design, the large, relatively flat interaction surface significantly complicated the task. Based on the analysis of the structure of the PD-1/PD-L1 complex, we have recently proposed the three likely hot spots [20], but only the structures reported in this study allowed to confirm the suitability of those for the design of small molecule inhibitors and defined particular interactions (pharmacophore) which should be explored. Importantly, these “hot spots” can likely be targeted by the compounds that not necessarily induce dimerization. Our study redefines the previously proposed sites and allows pinpointing PD-L1 residues important for the inhibitor binding with higher accuracy. As shown by the present crystal structures, BMS-202 and BMS-8 each target two of the previously described hot spots, which now may be treated as a single continuous interaction area. This target space consists of Tyr56, Met115, Ile116, Ala121 and Tyr123 forming an extended groove ideal for accommodating hydrophobic moieties (Figure S6). Presented herein new composite binding cleft does not exclude possible use of the previously described single “hot spots”, thereby creating novel possibilities for further inhibitor design.

**Figure 5:**
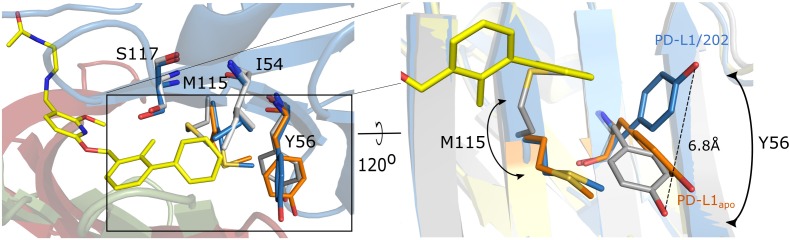
Conformational changes upon PD-L1 interaction with BMS-202 Overlay of apo-PD-L1 (orange; PDB 5C3T) and PD-L1 structures derived from PD-L1/PD-1 complex (gray; PDB 4ZQK) and PD-L1/BMS-202 complex (blue - chain A) demonstrates significant rearrangement of Tyr56 sidechain disposition upon BMS-202 binding compared to much less pronounced changes observed upon PD-1 binding (Note the T-stacking interaction of Tyr56 with the distal phenyl moiety of the inhibitor. Similar for BMS-8 containing structure (not shown). The rearrangement of Met115 sidechain is in turn more pronounced upon PD-1 binding compared to BMS-202 binding. Chain B of the PD-L1/BMS-202 dimer (green). PD-1 (red).

Interestingly, the inhibitor binding induces a small, but significant rearrangement of the sidechains at the surface of PD-L1 (compared to the apo-structure), which is partly different to that induced by the PD-1 interaction. In the case of the PD-1/PD-L1 complex, Tyr56 of PD-L1 is rotated and rearranged towards the PD-L1 core, while the Tyr56 sidechain ring in PD-L1/BMS-202 complex is considerably moved towards the inhibitor molecule thus creating a T-stacking interaction with the benzyl moiety of the latter (Figure 5). Similar rearrangement is observed upon BMS-8 binding. This dynamic adjustment in PD-L1 upon inhibitor binding provides further guidance for the rational design of small-molecule binders to PD-L1. Additionally, less pronounced adjustments include Ala18, Thr20, Met115, Ser117 and Tyr123 residues in the close vicinity of the inhibitor. Even though the compound does not impose significant changes in the overall backbone arrangement, minor rearrangements are visible in residues distant to the inhibitor binding site including Pro24, Asp61, His69, Glu71, Tyr81 and Arg113. These changes, however, are most likely caused by the dimerization rather than the small-molecule binding.

This study was performed using soluble distal extracellular IgG like domain of PD-L1 which served well to characterize protein surface accessible for targeting with small molecule inhibitors. At the cell surface, however, PD-L1 is constrained by being anchored at the lipid bilayer. Overlay of the structure of full length extracellular domain of PD-L1 containing both IgG like domains on BMS-202 induced dimer and docking such a complex at the lipid membrane demonstrates that dimerization is sterically feasible at the cell surface (same is true for BMS-8 containing dimer). This is owned to a long spacer between the membrane and a relatively rigid “core” of the extracellular domain of PD-L1, which linker allows enough flexibility for the core to form a dimer characterized by geometry observed in our structures (Figure S11A).

The proteins used in this study were produced in bacteria and as such lack glycosylation. We therefore evaluated if mammalian glycosylation could preclude BMS compound induced PD-L1 dimerization. The known glycosylation sites on PD-L1 are shown in Figure S11B and clearly do not sterically interfere with BMS-202 induced dimerization. This was expected since PD-L1 employs the same surface for dimerization and interaction with PD-1. Any glycosylation preventing dimerization would also preclude PD-1 binding.

Differential scanning fluorimetry employed here to evaluate the BMS compound selectivity between PD-L1 and PD-L2 demonstrated that both BMS-8 and BMS-202 are specific for PD-L1. No crystal structure of the human PD-L2 (hPD-L2) is available to date and only murine PD-L2 (mPD-L2) extracellular domain was crystallized in an apo form (PDB 3BOV) and in the complex with the murine PD-1 (mPD-1; PDB 3BP5). To speculate on the likely determinants of BMS compound specificity, we created a homology model of hPD-L2 based on mPD-L2 structures. Both proteins contain a tryptophan residue (Trp57 in hPD-L1 and Trp110 in mPD-L2) in the center of the PD-1 interacting surfaces. However, the sidechain of this residue in hPD-L1 is hidden inside hydrophobic core of the protein (does not contribute to the interaction with PD-1 or BMS-202), while the sidechain of corresponding tryptophan in mPD-L2 is exposed to the solvent in the apo structure and oriented towards the PD-1 core in mPD-1/mPD-L2 complex. Since the amino acid sequence identity of extracellular domains of hPD-L2 and mPD-L2 is 73% and both proteins contain tryptophan at equivalent position (Ala-Trp-Asp-Tyr sequence in both proteins), it is likely that the sidechain of Trp57 in hPD-L2 has the same conformation as the corresponding tryptophan in mPD-L2. The resulting different surface arrangement of hPD-L1 and hPD-L2 at the binding site would explain the specificity of BMS compounds for hPD-L1.

In conclusion, the presented data documents structural determinants guiding the recognition of small molecule inhibitors of PD-1/PD-L1 interaction by PD-L1. The unambiguous definition of the molecular “hot spots” at the surface of PD-L1 provides solid basis for future development of immunomodulating small molecules against cancer.

## MATERIALS AND METHODS

### Expression and purification of recombinant PD-L1, PD-L2 and PD-1

The gene encoding human PD-L1 (amino acids 18-134) was cloned into the pET-21b, the gene encoding human PD-L2 (20-220) was cloned into pET28a and that of human PD-1 (33-150, Cys93 exchanged to serine) into pET-24d. Proteins were expressed in the *E. coli* BL21 (DE3). Cells were cultured in LB at 37°C. The protein production was induced with 1 mM IPTG at OD_600_ of 1.0 and the cells were cultured for additional 5h. For hPD-1, after induction the temperature was lowered to 30°C. Proteins were expressed as inclusion bodies which were collected by centrifugation, washed twice with 50 mM Tris-HCl pH 8.0 containing 200 mM NaCl, 0.5% Triton X-100, 10 mM EDTA and 10 mM 2-mercaptoethanol and once more with the same buffer with no detergent. The inclusion bodies were stirred overnight in 50 mM Tris pH 8.0 containing 6M GuHCl, 200 mM NaCl and 10 mM 2-mercaptoethanol. Solubilized fraction was clarified by high speed centrifugation. hPD-L1 and hPD-L2 were refolded by drop-wise dilution into 0.1 M Tris pH 8.0 containing 1 M L-Arg hydrochloride, 0.25 mM oxidized glutathione and 0.25 mM reduced glutathione for hPD-L1 and 0.1 M Tris pH 8.5 containing 1 M NDSB201, 0.2 M NaCl, 5 mM cysteamine and 0.5 mM cystamine for hPD-L2. hPD-1 was refolded in similar manner in 0.1 M Tris pH 8.0 containing 0.4 M L-Arg hydrochloride, 2 mM EDTA, 5 mM cystamine and 0.5 mM cysteamine. After refolding, the proteins were dialyzed 3 times against 10 mM Tris pH 8.0 containing 20 mM NaCl, and purified by size exclusion chromatography on Superdex 75 (GE Healthcare) in 10 mM Tris pH 8.0 containing 20 mM NaCl. The purity and protein folding were evaluated by SDS-PAGE and NMR, respectively.

### Analytical size-exclusion chromatography

The oligomeric state of tested proteins was analyzed by size exclusion chromatography. Superdex 75 10/30 HR (GE Healthcare) was equilibrated with PBS pH 7.4 and calibrated using globular proteins of known molecular weight. Approximate molecular weight of apo-PD-L1 and PD-L1-small molecule complex (3:1 compound : protein molar ratio) were estimated using the calibration curve.

### Differential scanning fluorimetry (DSF)

DSF analysis was performed according to Niesen and colleagues [26]. In brief PD-L1 and PD-L2 (both 12.5 μM) were incubated alone, with BMS-202 or BMS-8 (both at 37.5 μM) in the presence of SYPRO Orange Dye (Life Technologies). Constant temperature gradient of 0.2°C/min was applied and changes in fluorescence were monitored using real time thermocycler (BioRad). Melting temperature (T_m_) was estimated from the first derivative of fluorescence intensity as a function of temperature.

### NMR methods

Uniform ^15^N labeling was obtained by expressing the protein in the M9 minimal medium containing ^15^NH_4_Cl as the sole nitrogen source. Unlabeled proteins were prepared as for crystallization. For NMR measurements the buffer was exchanged by gel filtration to PBS pH 7.4 (PD-L1) or 25 mM sodium phosphate containing 100 mM NaCl pH 6.4 (PD-1). 10% (v/v) of D_2_O was added to the samples to provide lock signal. All spectra were recorded at 300K using a Bruker Avance 600 MHz spectrometer.

Binding of the compounds was analyzed by titrating the ^15^N-labeled PD-L1 (0.3 mM) and recording the ^1^H-^15^N HMQC spectra prior and after addition of the compound.

The ability of tested compounds to dissociate PD-L1 / PD-1 was evaluated using AIDA [25]. ^15^N-labeled PD-1 (0.2 mM) was slightly overtitrated with unlabeled PD-L1. Compound was aliquoted into the resulting mixture. During the experiment the ^1^H-^15^N signals were monitored by HMQC experiment.

Changes in the oligomeric state of PD-L1 in the presence of tested compounds were monitored by titration of unlabeled PD-L1 (0.3 mM) while recording ^1^H spectra prior and after addition of the compound. The approximate molecular weights of protein populations present in the sample were determined by analyzing the linewidth (relaxation time) of well separated NMR signals.

### Crystallization of PD-L1 in complex with BMS-202 and BMS-8

Purified PD-L1 in 10 mM Tris pH 8.0 containing 20 mM NaCl was mixed with BMS-202 or BMS-8 at 1:1 molar ratio. The complex was concentrated to 8 mg/ml. Sitting drop vapor diffusion setup and commercially available buffer sets were used to screen for crystallization conditions. Initially obtained crystals were optimized according to art. Diffraction-quality crystals of PD-L1/BMS-202 complex were obtained at room temperature from 0.01 M Tris pH 8.5 containing 0.30 M sodium chloride and 27% (w/v) PEG 4000 while those of PD-L1/BMS-8 complex from 0.2 M ammonium formate and 20% (w/v) PEG 3350.

### Structure determination and refinement

Crystals were flash-cooled in liquid nitrogen without additional cryoprotection. The diffraction data was collected at the Helmholtz Centrum 14.1 beamline at BESSY (Berlin, Germany) [34]. The data were indexed and integrated using XDS [35–36] and scaled and merged using Scala [37]. Molecular replacement was calculated using Phaser [38]. The structure of the recently solved human PD-L1 (PDB 5C3T) was used as a probe [20]. The protein models were manually built in the resulting electron density maps using Coot [39]. Restrained refinement was performed using Phenix [40] and Refmac 5.0 [41]. Five percent of the reflections were used for cross-validation analysis. The behavior of R_free_ was employed to monitor the refinement strategy. Inhibitor model and restraints were prepared in eLBOW [42] and introduced into the model at advanced stage of refinement when electron density describing the ligand was clearly visible. Water molecules were added using Coot and manually inspected. Molecular graphics was prepared with PyMOL (http://www.pymol.org/).

### Synthesis of compounds BMS-8, -37, -202 and BMS-242

2-methoxy-6-[(2-methyl-3-phenylphenyl)methoxy]pyridine-3-carbaldehyde, 3-bromo-4-((2-methyl-[1, 1′-biphenyl]-3-yl)methoxy)benzaldehyde and the final compounds were prepared according to procedures described in BMS patent [18] with minor modifications (Figure S12). All reagents were obtained from Sigma Aldrich and used without additional purification. NMR spectra were recorded on Bruker Avance 300 or 600 MHz spectrometers (Figures S13-S16). All chemical shifts (δ) are reported in ppm and coupling constants (J) in Hz. The identity and purity of all compounds was additionally analyzed by high-resolution mass spectrometry (HRMS) and HPLC.

### Accession codes

Coordinates and structure factors were deposited in the Protein Data Bank with accession numbers 5J89 (BMS-202) and 5J8O (BMS-8).

## SUPPLEMENTARY MATERIAL FIGURES



## References

[1] Mahoney KM, Paul D, Rennert PD, Freeman JG (2015). Combination cancer immunotherapy and new immunomodulatory targets. Nat Rev Drug Disc.

[2] Topalian SL, Drake CG, Pardoll DM (2015). Immune checkpoint blockade: a common denominator approach to cancer therapy. Cancer Cell.

[3] Sharma P, Allison JP (2015). The future of immune checkpoint therapy. Science.

[4] Shin DS, Ribas A (2015). The evolution of checkpoint blockade as a cancer therapy: what's here, what's next?. Curr Opi Imunn.

[5] Mullard A (2015). FDA approves first immunotherapy combo. Nat. Rev. Drug Discov.

[6] Ohaegbulam KC, Assal A, Lazar-Molnar E, Yao Y, Zang XX (2015). Human cancer immunotherapy with antibodies to the PD-1 and PD-L1 pathway. Trends Mol. Med.

[7] Dömling A, Holak TA (2014). Programmed death-1: therapeutic success after more than 100 years of cancer immunotherapy. Angew Chem Int Ed.

[8] Sunshine J, Taube JM (2015). PD-1/PD-L1 inhibitors. Curr Opin Pharmacol.

[9] Brahmer JR, Tykodi SS, Chow LQM, Hwu WJ, Topalian SL, Hwu P, Drake CG, Camacho LH, Kauh J, Odunsi K, Pitot C, Hamid O, Bhatia S (2012). Safety and activity of anti-PD-L1 antibody in patients with advanced cancer. N Engl J Med.

[10] Brahmer JR, Rizvi NA, Lutzky J, Khleif S, Blake-Haskins A, Li X, Robbins PB, Vasselli J, Ibrahim RA, Antonia SJ (2014). J Clin Oncol.

[11] Herbst RS, Soria JC, Kowanetz M, Fine GD, Hamid O, Gordon MS, Sosman JA, McDermott DF, Powderly JD, Gettinger SN, Kohrt HE, Horn L, Lawrence DP (2014). Predictive correlates of response to the anti-PD-L1 antibody MPDL3280A in cancer patients. Nature.

[12] Powles T, Eder JP, Fine GD, Braiteh FS, Loriot Y, Cruz C, Bellmunt J, Burris HA, Petrylak DP, Teng SL, Shen X, Boyd Z, Hegde PS (2014). MPDL3280A (anti-PDL1) treatment leads to clinical activity in metastatic bladder cancer. Nature.

[13] Taube JM, Klein A, Brahmer JR, Xu H, Pan X, Kim JH, Chen L, Pardoll DM, Topalian SL, Anders RA (2014). Association of PD-1, PD-1 ligands, and other features of the tumor immune microenvironment with response to anti-PD-1 therapy. Clin Cancer Res.

[14] Sasikumar PGN, Ramachandra M, Vadlamani SK, Vemula RK, Satyam LK, Subbarao K, Shrimali RK, Kandepu S Immunosuppression modulating compounds. Aurigene Discovery Technologies Limited.

[15] Sasikumar PGN, Ramachandra M, Naremaddepalli SSS (2013). Peptidomimetic compounds as immunomodulators. Aurigene Discovery Technologies Limited.

[16] Miller MM, Mapelli C, Allen MP, Bowsher MS, Boy KM, Gillis EP, Langley DR, Mull E, Poirier MA, Sanghvi N, Sun LQ, Tenney DJ, Yeung KS, Zhu J (2014). Macrocyclic inhibitors of the PD-1/PD-L1 and CD80(B7-1)/PD-L1 protein/protein interactions. Bristol-Myers Squibb Company.

[17] Sasikumar PGN, Ramachandra M, Naremaddepalli SSS (2015). 1,2,4-Oxadiazole Derivatives as Immunomodulators. Aurigene Discovery Technologies Limited.

[18] Chupak LS, Zheng X (2015). Compounds useful as immunomodulators. Bristol-Myers Squibb Company.

[19] Lin DY, Tanaka Y, Iwasaki M, Gittis AG, Su HP, Mikami B, Okazaki T, Honjo T, Minato N, Garboczi DN (2008). The PD-1/PD-L1 complex resembles the antigen-binding Fv domains of antibodies and T cell receptors. Proc Natl Acad Sci USA.

[20] Zak KM, Kitel R, Przetocka S, Golik P, Guzik K, Musielak B, Dömling AS, Dubin G, Holak TA (2015). Structure of the Complex of Human Programmed Death-1 (PD-1) and Its Ligand PD-L1. Structure.

[21] Shuker SB, Hajduk PJ, Meadows RP, Fesik SW (1996). Discovering high-affinity ligands for proteins: SAR by NMR. Science.

[22] Stoll R, Renner C, Hansen S, Palme S, Klein C, Belling A, Zeslawski W, Kamionka M, Rehm T, Mühlhahn P, Schumacher R, Hesse F, Kaluza B (2001). Chalcone derivatives antagonize interactions between the human oncoprotein MDM2 and p53. Biochemistry.

[23] D'silva L, Ozdowy P, Krajewski M, Rothweiler U, Singh M, Holak TA (2005). Monitoring the effects of antagonists on protein-protein interactions with NMR spectroscopy. J Amer Chem Soc.

[24] Bista M, Kowalska K, Janczyk W, Dömling A, Holak TA (2009). Robust NMR screening for lead compounds using tryptophan-containing proteins. J Amer Chem Soc.

[25] Krajewski M, Rothweiler U, D'silva L, Majumdar S, Klein C, Holak TA (2007). An NMR-based antagonist induced dissociation assay for targeting the ligand-protein and protein-protein interactions in competition binding experiments. J Med Chem.

[26] Niesen FH, Berglund H, Vedadi M (2007). The use of differential scanning fluorimetry to detect ligand interactions that promote protein stability. Nature Protocols.

[27] Matulis D, Kranz JK, Salemme FR, Todd MJ (2005). Thermodynamic stability of carbonic anhydrase: measurements of binding affinity and stoichiometry using ThermoFluor. Biochemistry.

[28] de Beer TAP, Berka K, Thornton JM, Laskowski RA (2014). PDBsum additions. Nucleic Acids Res.

[29] Cheng X, Veverka V, Radhakrishnan A, Waters LC, Muskett FW, Morgan SH, Huo J, Yu C, Evans EJ, Leslie AJ, Griffiths M, Stubberfield C, Griffin R (2013). Structure and interactions of the human programmed cell death 1 receptor. J Biol Chem.

[30] Clackson T, Wells JA (1995). A Hot Spot of Binding Energy in a Hormone-Receptor Interface. Science.

[31] Arkin MR, Wells JA (2004). Small Molecule Inhibitors of Protein-Protein Interactions: Progressing Towards the Dream. Nature Rev. Drug Disc.

[32] Arkin MR, Tang Y, Wells JA (2014). Small-Molecule Inhibitors of Protein-Protein Interactions: Progressing toward the Reality. Chem. Biol.

[33] Chen Y., Liu P., Gao F., Cheng H., Qi J., Gao GF (2010). A dimeric structure of PD-L1: functional units or evolutionary relics?. Protein Cell.

[34] Mueller U, Förster R, Hellmig M, Huschmann FU, Kastner A, Malecki P, Pühringer S, Röwer M, Sparta K, Steffien M, Ühlein M, Wilk P, Weiss MS (2015). The macromolecular crystallography beamlines at BESSY II of the Helmholtz-Zentrum Berlin: Current status and perspectives. Eur Phys J Plus.

[35] Krug M, Weiss MS, Heinemann U, Mueller U (2012). XDSAPP: a graphical user interface for the convenient processing of diffraction data using XDS. J Appl Cryst.

[36] Kabsch W (2010). XDS. Acta Crystallogr D Biol Crystallogr.

[37] Evans PR (2010). Scaling and assessment of data quality. Acta Crystallogr D Biol Crystallogr.

[38] McCoy AJ, Grosse-Kunstleve RW, Adams PD, Winn MD, Storoni LC, Read AJ (2007). Phaser crystallographic software. J Appl Cryst.

[39] Emsley P, Lohkamp B, Scott WG, Cowtan K (2010). Features and development of Coot. Acta Crystallogr D Biol Crystallogr.

[40] Adams PD, Afonine PV, Bunkóczi G, Chen VB, Davis IW, Echols N, Headd JJ, Hung LW, Kapral GJ, Grosse-Kunstleve RW, McCoy AJ, Moriarty NW, Oeffner R (2010). PHENIX: a comprehensive Python-based system for macromolecular structure solution. Acta Crystallogr D Biol Crystallogr.

[41] Murshudov GN, Skubak P, Lebedev AA, Pannu NS, Steiner RA, Nicholls RA, Winn MD, Long F, Vagin AA (2011). REFMAC5 for the refinement of macromolecular crystal structures. Acta Crystallogr D Biol Crystallogr.

[42] Moriarty NW, Grosse-Kunstleve RW, Adams PD (2009). Electronic Ligand Builder and Optimization Workbench (eLBOW): a tool for ligand coordinate and restraint generation. Acta Crystallogr D Biol Crystallogr.

