# Supplementary material for: Structural basis for small molecule targeting of the programmed death ligand 1 (PD-L1)
Source: Oncotarget. 2016 Apr 13;7(21):30323–35. doi: 10.18632/oncotarget.8730 (PMC5058683; doi:10.18632/oncotarget.8730)
Supplement: Supplementary file 1 [file oncotarget-07-30323-s001.pdf]

Structural basis for small molecule targeting of the programmed death ligand 1 (PD-L1)

Supplementary Material

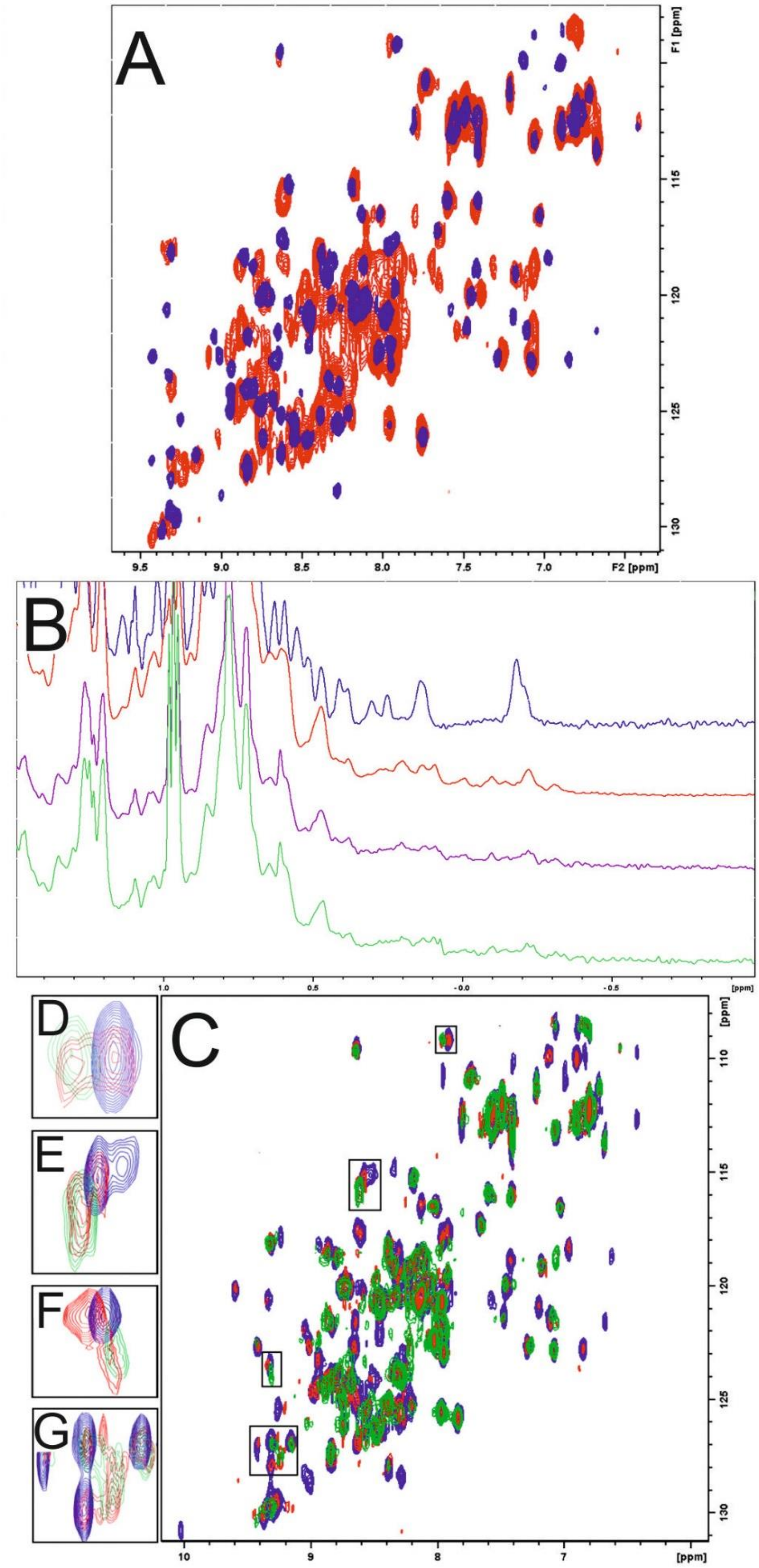

Figure S1

NMR monitored titration of hPD-L1 with BMS-202. In each case the molar ratio of protein : compound is given. (**A**) ^{1}H-^{15}N HMQC monitored overtitration indicates **BMS-202** interaction with hPD-L1. Spectra of hPD-L1 in absence (blue) and presence of equimolar **BMS-202** (red) are shown. Peak shifts indicate compound binding, linewidth broadening indicates protein dimerization. (**B**) ^{1}H NMR titration suggests **BMS-202** induced PD-L1 dimer formation. hPD-L1 (blue), hPD-L1: **BMS-202** molar ratios 10:1 (red), 4:1 (purple), and 1:2 (green). Linewidth broadening associated loss of resonance peaks in the aliphatic region (shown) indicates significant increase in the molecular weight of the complex which may not be explained by interaction with **BMS-202** only, thus suggesting **BMS-202** induced dimerization. (**C**) same as in panel A, but at intermediate PD-L1:**BMS-202** molar ratios: PD-L1 only (blue), 5:1 (red), 4:1 (green); (**D**)-(**G**) Enlarged fragments of spectra shown in panel c. Two separate sets of ^{1}H-^{15}N HMQC resonances are observed in the intermediate stages of titration, one corresponding to free PD-L1 and the other to the PD-L1 bound to the **BMS-202**. This demonstrates that the **BMS-202**/PD-L1 complex is long lived on the NMR chemical shift time scale indicating strong binding with K_D<1 µM.

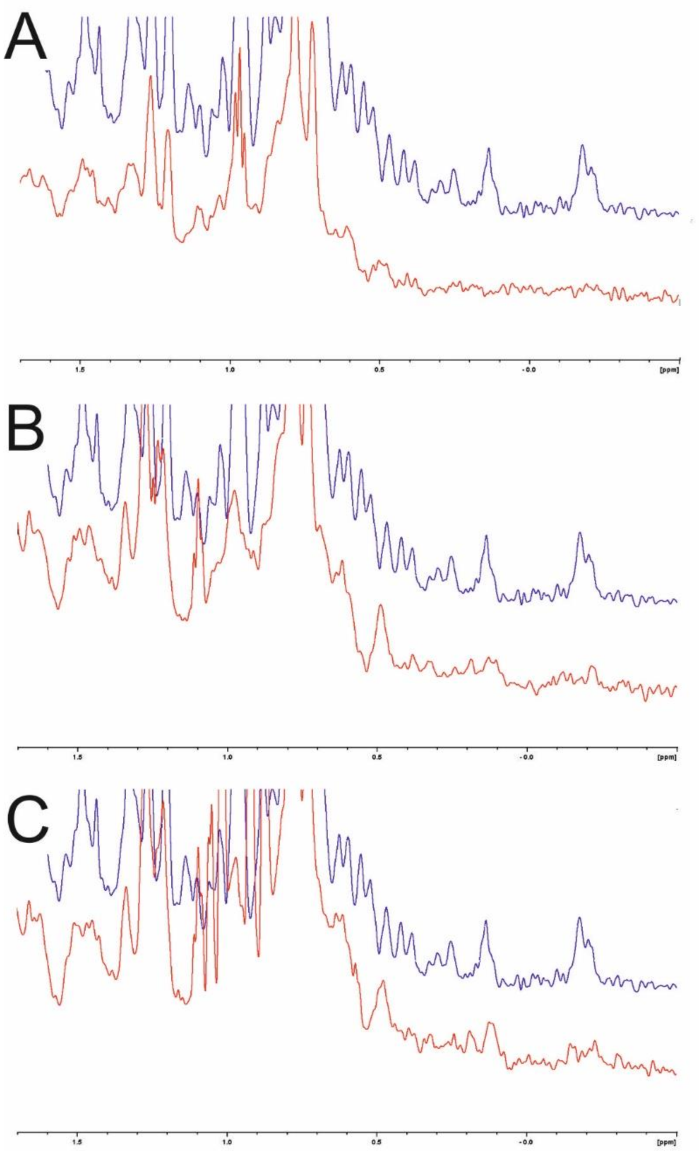

Figure S2

BMS-8, -37 and -242 induce dimerization of PD-L1 in solution. The aliphatic region of the ^{1}H NMR spectra is shown. Linewidth broadening associated loss of the NMR signals at ca. -0.2 ppm indicates a significant increase in the molecular weight of the complex, suggesting compound-induced dimerization (compare Supplementary Figure 1). (**A**) PD-L1 (blue), PD-L1:**BMS-8** at 4:1 molar ratio (red) (**B**) PD-L1 (blue), PD-L1: **BMS-37** at 1:1 molar ratio (red). (**C**) PD-L1 (blue), PD-L1: **BMS-242** at 1:1 molar ratio (red).

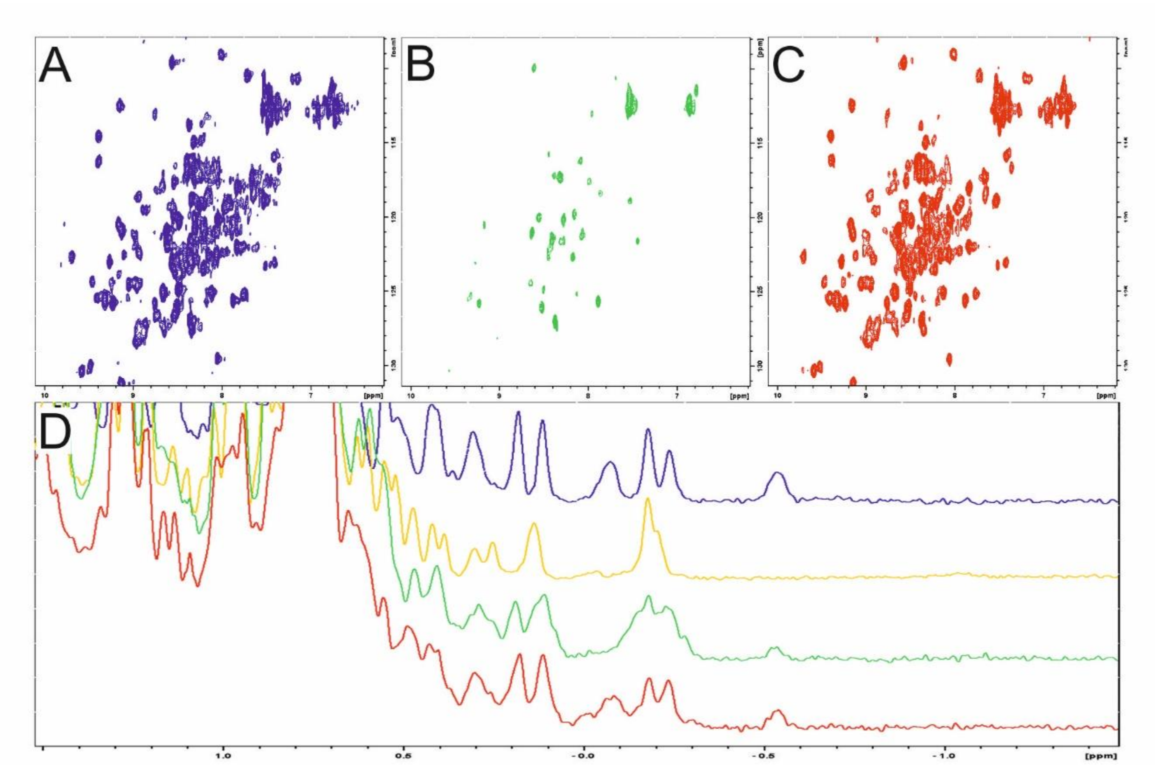

Figure S3

BMS-202 dissociates a preformed PD-1/PD-L1 complex. ^{1}H-^{15}N HMQC spectra are shown in panels A, B and C. (**A**) ^{15}N labeled PD-1. (**B**) Complex of ^{15}N labeled PD-1 and unlabeled PD-L1. Linewidth broadening observed as loss of resonance peaks (compare panels A and B) indicates increased relaxation time associated with complex formation. (**C**) same as in panel B but after addition of **BMS-202** at equimolar ratio. Decrease in relaxation time (compare panels C and B) evidences decrease of the molecular weight of labeled protein containing specie owned to ^{15}N-PD-1/^{14}N-PD-L1 complex dissociation by **BMS-202**. (**D**) Same as in panels A-C, but using unlabeled proteins and monitored by ^{1}H NMR. The aliphatic region of ^{1}H NMR spectrum is shown. At ca. -0.2 ppm signals from PD-1 (blue) and PD-L1 (yellow) overlay in the spectrum of PD-1/PD-L1 complex (green). Upon addition of equimolar **BMS-202** to preformed PD-1/PD-L1 complex the signal of PD-L1 is lost (red) due to compound induced dissociation of the complex and PD-L1 dimer formation.

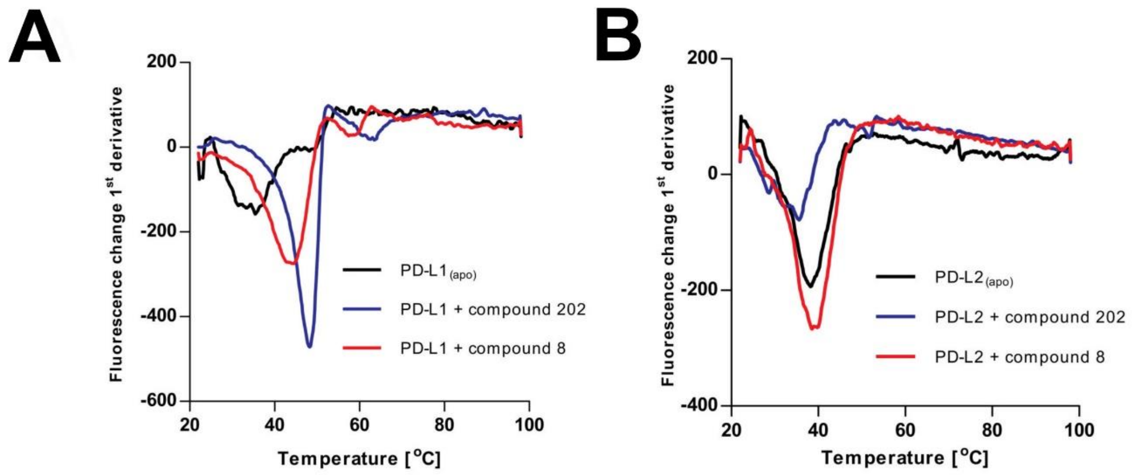

Figure S4

BMS-202 and -8 induce thermal stabilization of PD-L1 but not PD-L2. Thermal unfolding of the protein was monitored by Differential Scanning Fluorimetry. First derivatives of temperature dependence of fluorescence intensity are shown. A significant compound induced shift in melting temperature is observed for hPD-L1 (**A**) but not hPD-L2 (**B**).

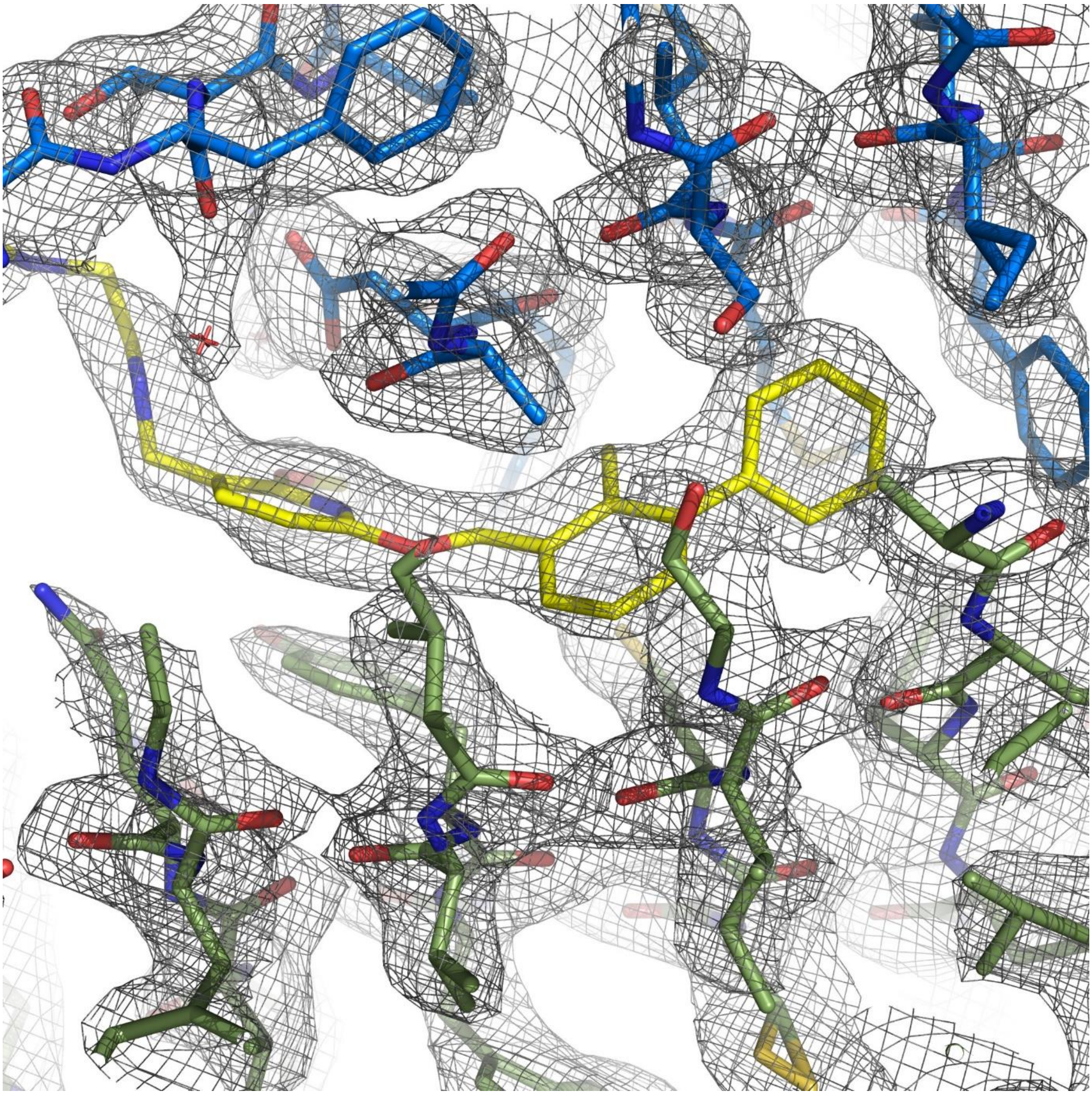

Figure S5

Quality of electron density map for BMS-202/PD-L1 complex structure. Example 2Fo-Fc map contoured at 1σ shows continuous, well interpretable electron density describing the inhibitor and the surrounding residues within the binding site.

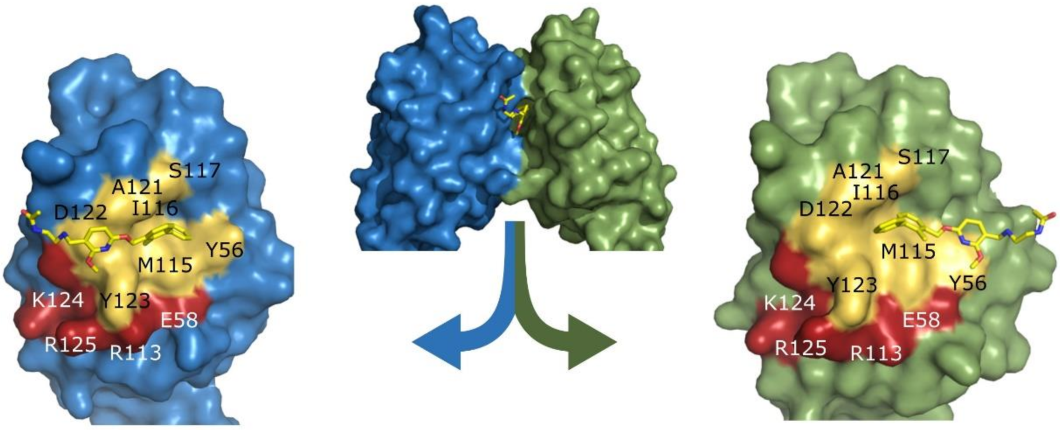

Figure S6

"Hot spots" on the surface of PD-L1 suitable for targeting with low molecular weight inhibitors. To depict the **BMS-202** interaction surface defined in this study (gold, corresponding residues labeled black) the dimer was split into monomers (note that this does not imply that **BMS-202** is capable of interacting with a single molecule of PD-L1). Previously described additional sites likely suitable for small molecule targeting are depicted in red with corresponding residues labeled white. Comparable sites are defined by **BMS-8** containing structure (not shown).

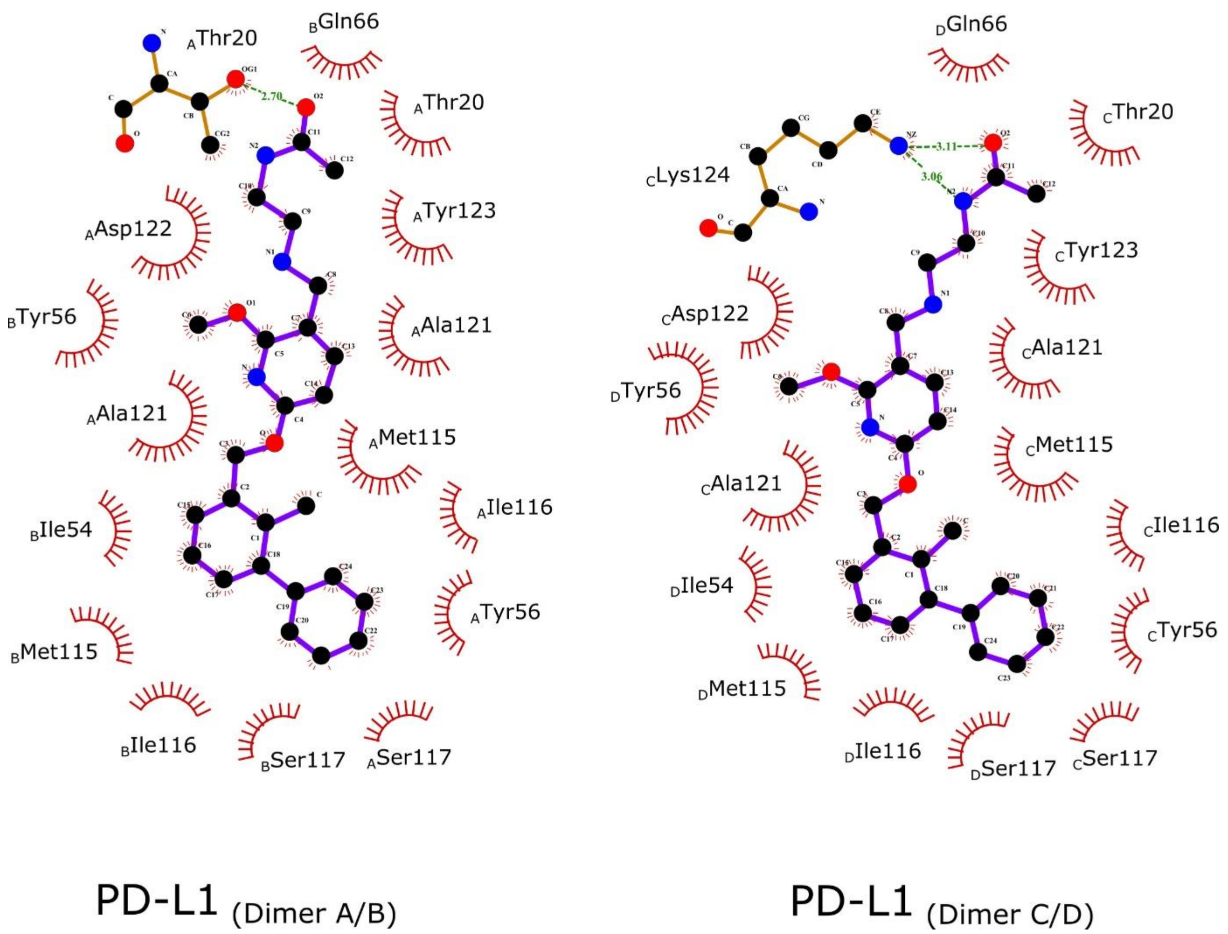

Figure S7

Schematic representation of BMS-202 binding site within PD-L1 dimer. Two inhibitor molecules contained in the asymmetric unit and protein residues contributing hydrogen bonds are shown in stick representation (CPK color coding). Other residues constituting the binding site are depicted as brown arches. Hydrogen bonds are shown as green dotted lines. Note that the extended *N*-(2-aminoethyl)acetamide moiety of the inhibitor contributes different interactions in A/B and C/D dimers.

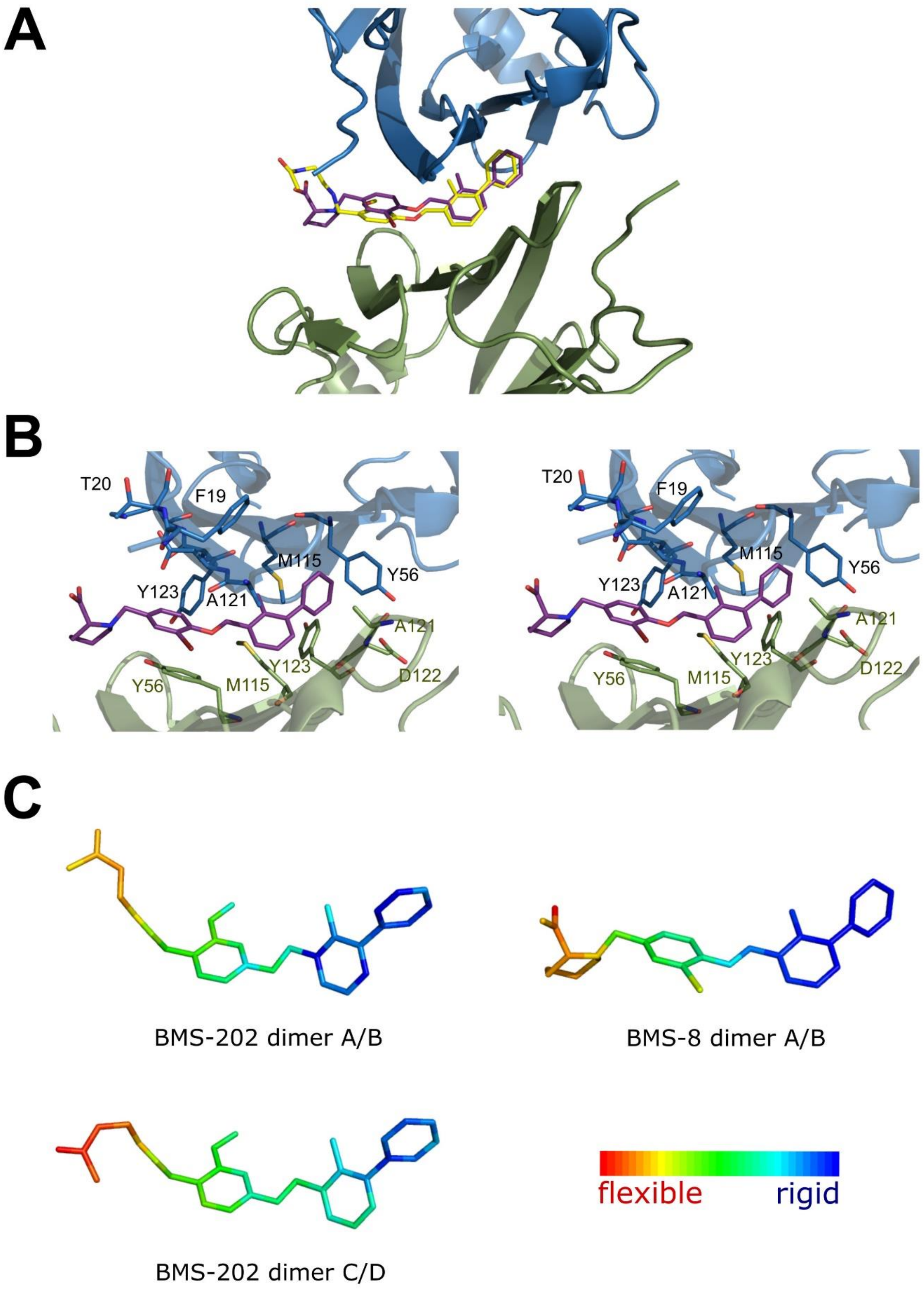
A
B
T20
F19
M115
Y123
A121
Y56
A121
Y123
D122
Y56
M115
T20
F19
M115
Y123
A121
Y56
A121
Y123
D122
Y56
M115
C
BMS-202 dimer A/B
BMS-8 dimer A/B
BMS-202 dimer C/D
flexible
rigid

Figure S8

(A) Overlay of PD-L1/**BMS-8** and PD-L1/**BMS-202** structures demonstrates that **BMS-8** (violet) and **BMS-202** (yellow) assume comparable conformations, occupy identical binding pocket and induce structurally comparable dimerization of PD-L1. Color coding as in Figure 2B. **(B)** Detailed interactions of **BMS-8** at the binding cleft of PD-L1. **BMS-8** binds at a hydrophobic cavity formed upon PD-L1 dimerization. Color coding as in Figure 2B. **(C)** Alternative conformations and thermal flexibility of **BMS-202** and **BMS-8** within the PD-L1 dimer**.** B-factors characterizing particular atoms are shown in color scale. Low B-factors describe more rigid fragments of the molecule. Note the thermal flexibility (high B-factors) and different orientations of the *N*-(2-aminoethyl)acetamide moiety (left-hand side) in the two dimers within PD-L1/**BMS-202** structure.

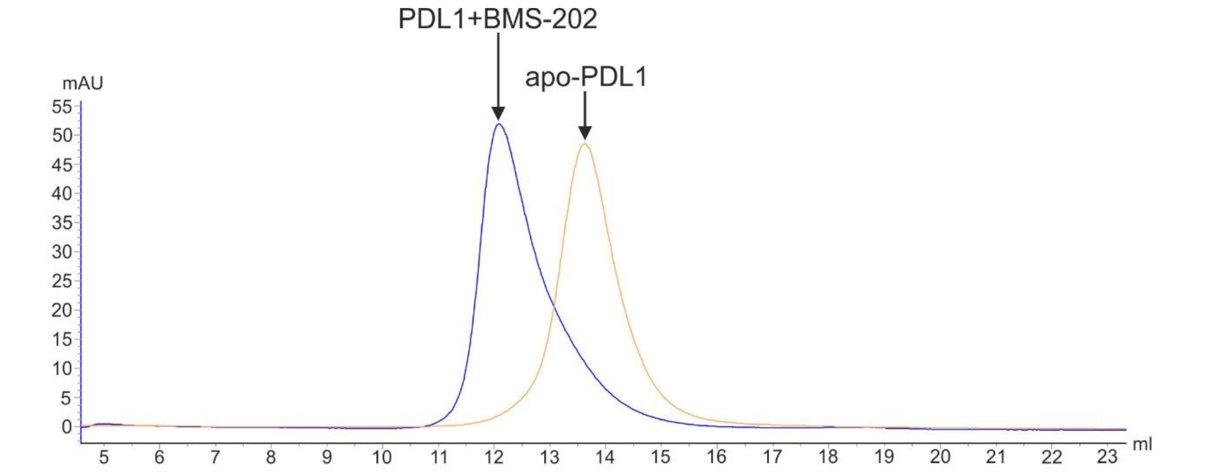

Figure S9

BMS-202 induces dimerization of PD-L1 in solution. Gel filtration chromatograms of PD-L1 in the presence and absence of **BMS-202** are shown. The complex exhibits shorter retention time indicating increased molecular weight compared to apo-PD-L1. Estimated molecular weights indicate that apo-PD-L1 elutes as a monomer whereas **BMS-202**/PD-L1 complex contains two molecules o PD-L1.

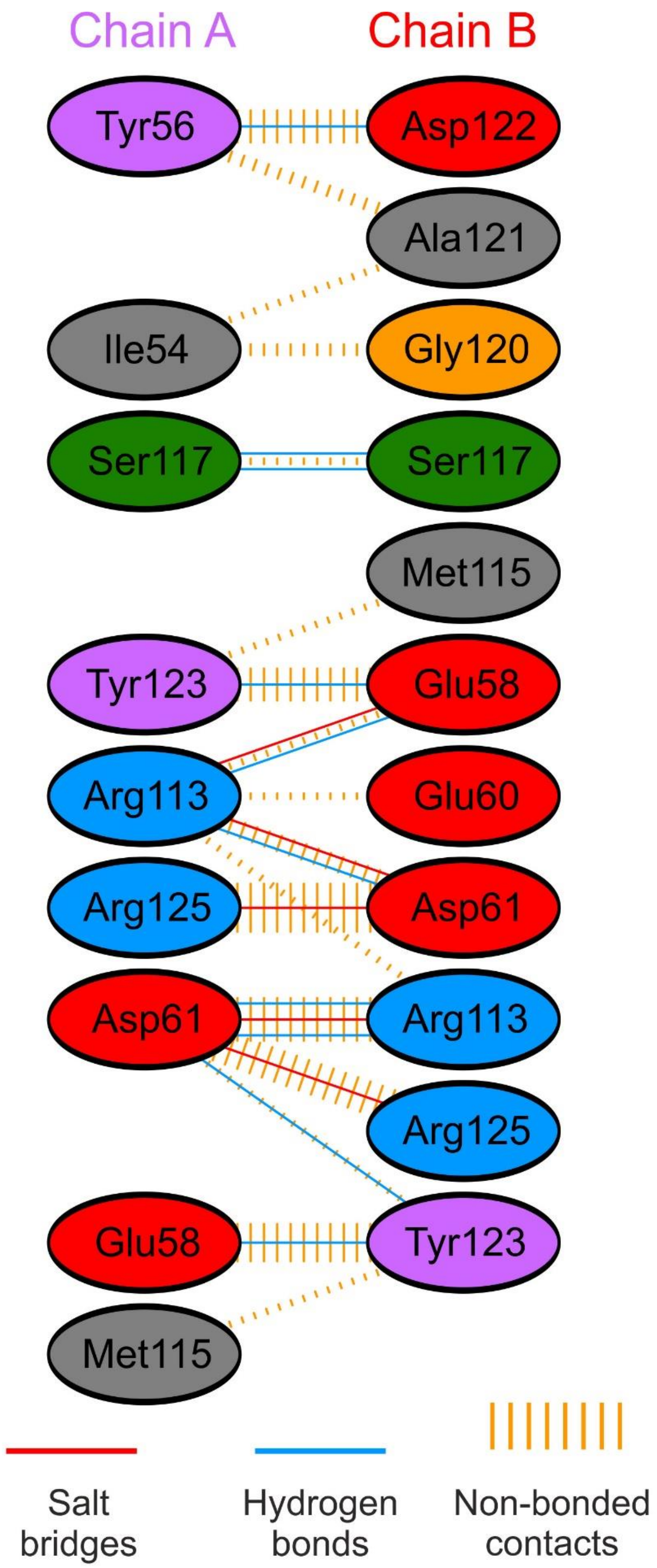
Chain A
Chain B
Tyr56
Asp122
Ala121
Ile54
Gly120
Ser117
Ser117
Met115
Tyr123
Glu58
Arg113
Glu60
Arg125
Asp61
Asp61
Arg113
Arg125
Glu58
Tyr123
Met115
Salt bridges
Hydrogen bonds
Non-bonded contacts

Figure S10

Schematic representation of protein-protein interactions within BMS-202 induced dimer of PD-L1 as observed in the crystal structure (A/B dimer; comparable interactions are observed in C/D dimer – not shown). Ellipsoids representing amino acids are colored according to the properties of their sidechains: positively charged (blue), negatively charged (red), polar uncharged (green), hydrophobic (grey), containing aromatic rings (purple) and glycine (yellow).

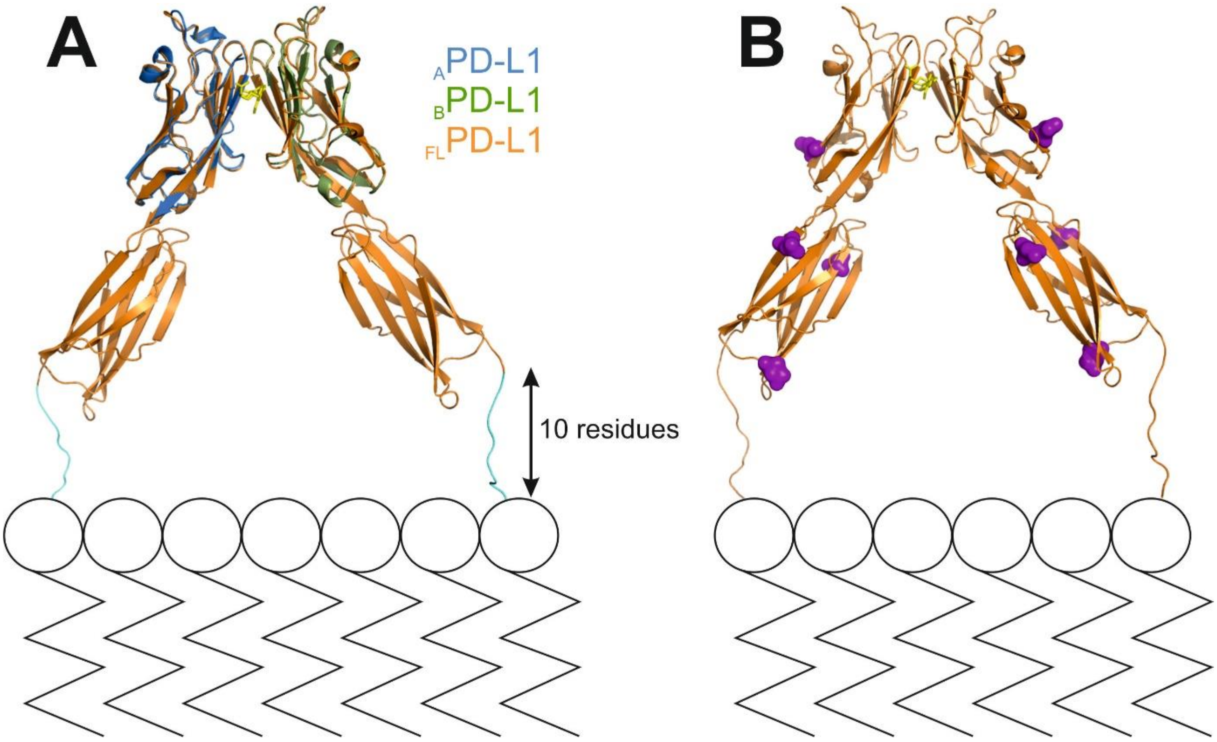

Figure S11

Model of BMS-202 induced dimerization of PD-L1 at the cell surface. (**A**) The structure of full length extracellular domain of PD-L1 (orange; PDB 3BIK) was overlaid on the structure of **BMS-202** (yellow; identical for **BMS-8** (not shown)) induced PD-L1 dimer (model A – blue, model B – green). Ten residue stalk (light blue) connecting the structured extracellular domain of PD-L1 and the membrane, which is not defined in the crystal structure, but present in the protein sequence (Uniprot Q9NZQ7), was computationally added. The resulting complex was docked at the lipid bilayer demonstrating that **BMS-202** induced PD-L1 dimerization defined in this study for soluble extracellular domain is sterically compatible with the physiological, membrane bound character of PD-L1. (**B**) Known glycosylation sites (purple) are depicted on a structural model of **BMS-202** induced PD-L1 dimer (same as in panel A). Note that the glycosylation sites (residues 35, 192, 200, 219) are located far from the dimer interface and therefore glycosylation is unlikely to interfere with **BMS-202** induced dimerization.

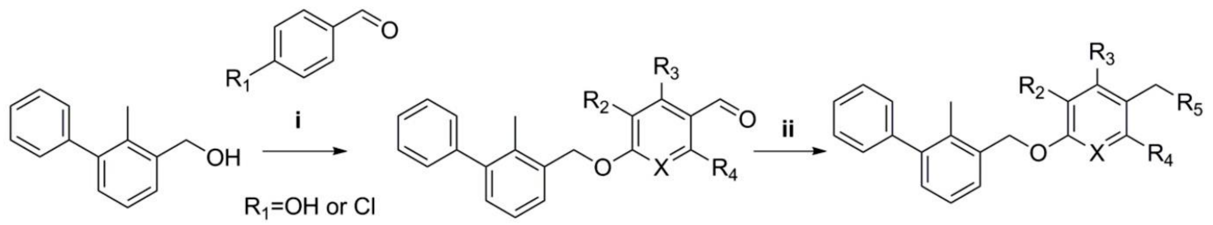

1a, 1b, 1c   2a, 2b, 2c, 2c1

No	R_2	R_3	R_4	X	R_5
1a	-H	-H	$-OCH_3$	N	-
1b	-Br	-H	-H	C	-
1c	-H	$-OCH_3$	$-OCH_3$	C	-
2a	-H	-H	$-OCH_3$	C	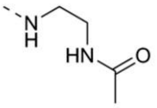
2b	-Br	-H	-H	C	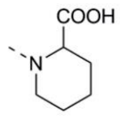
2c	-H	$-OCH_3$	$-OCH_3$	C	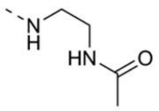
2c1	-H	$-OCH_3$	$-OCH_3$	C	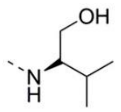

Figure S12

The general synthesis pathway of BMS compounds ([2-methyl-3-biphenylyl]methanol derivatives) evaluated in this study. i. PPh_3, DIAD or $Pd(OAc)_2$ or *tert*-butyl XPhos if R_1 is OH or Cl, respectively, ii. appropriate amine (R_5), $NaBH_3CN$, AcOH, DMF.

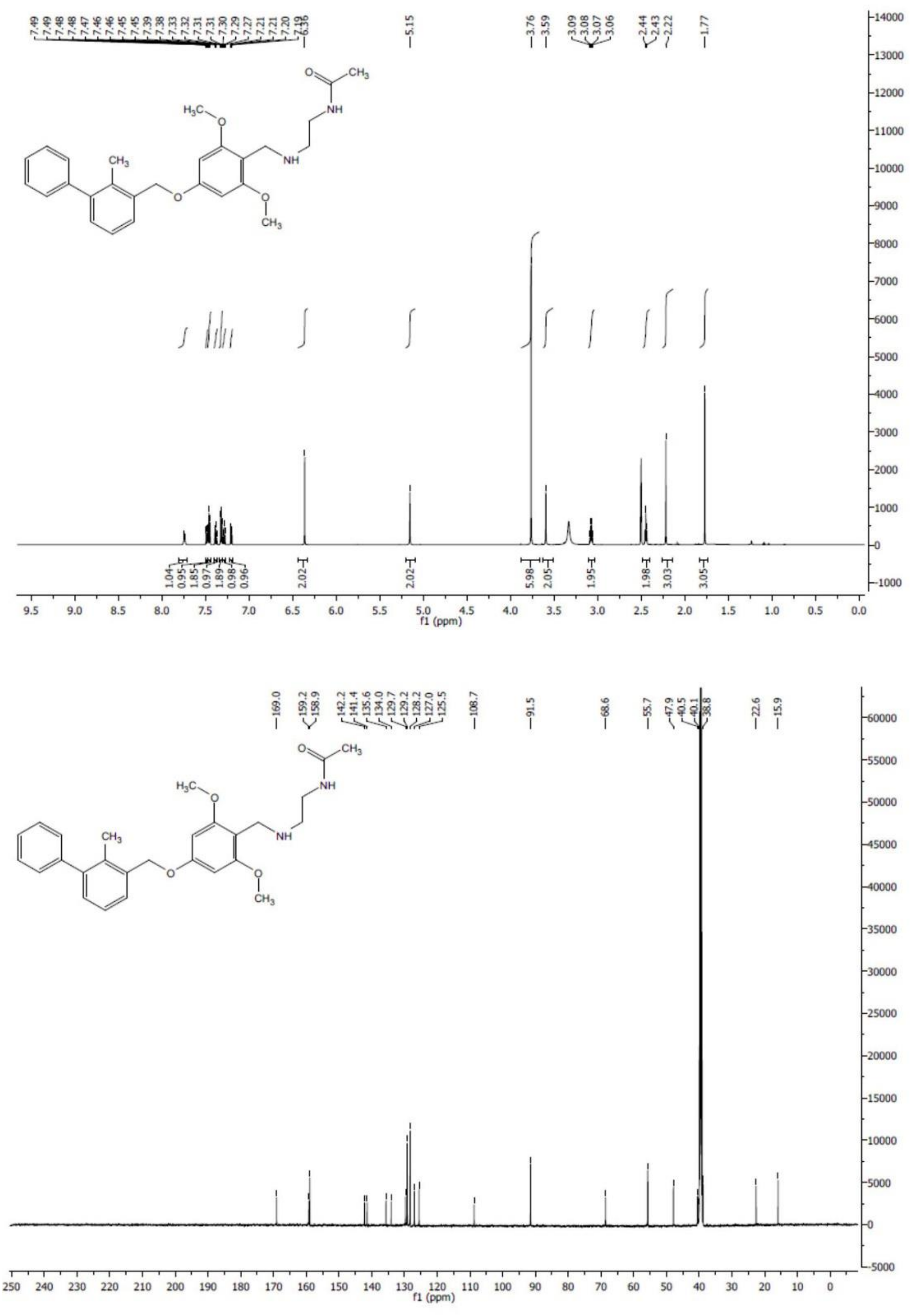

Figure S13

BMS-37 analysis. ^{1}H NMR (upper panel) and ^{13}C NMR (bottom panel).

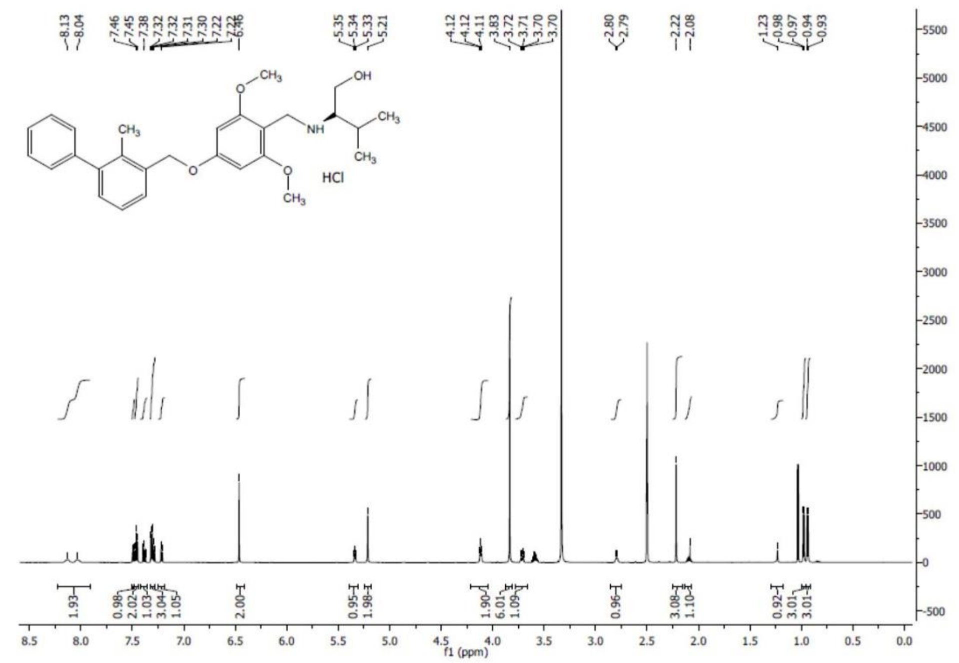

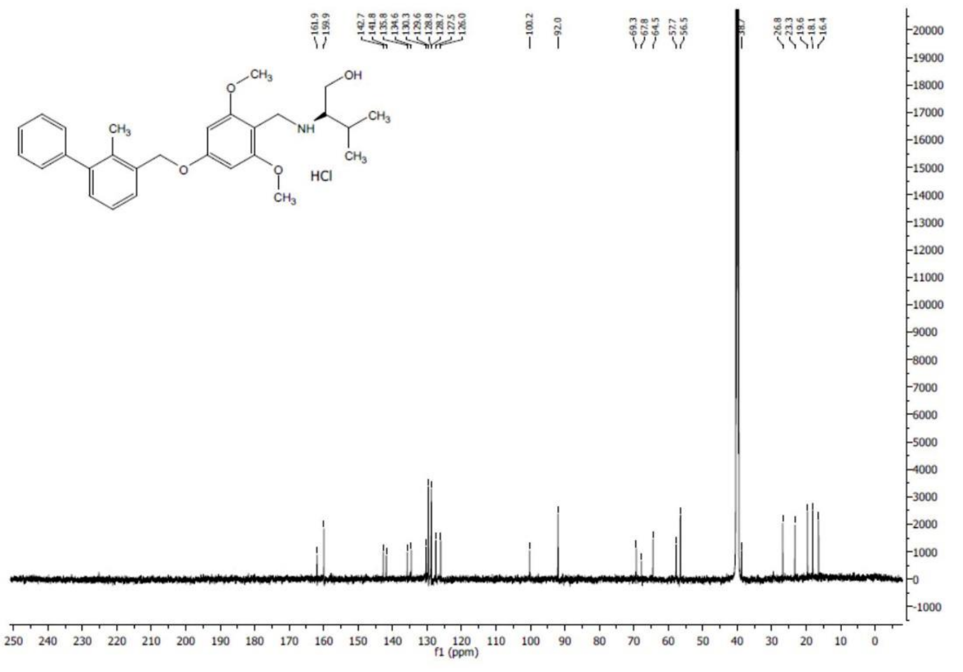

Figure S14

BMS-242 analysis. ^{1}H NMR (upper panel) and ^{13}C NMR (bottom panel).

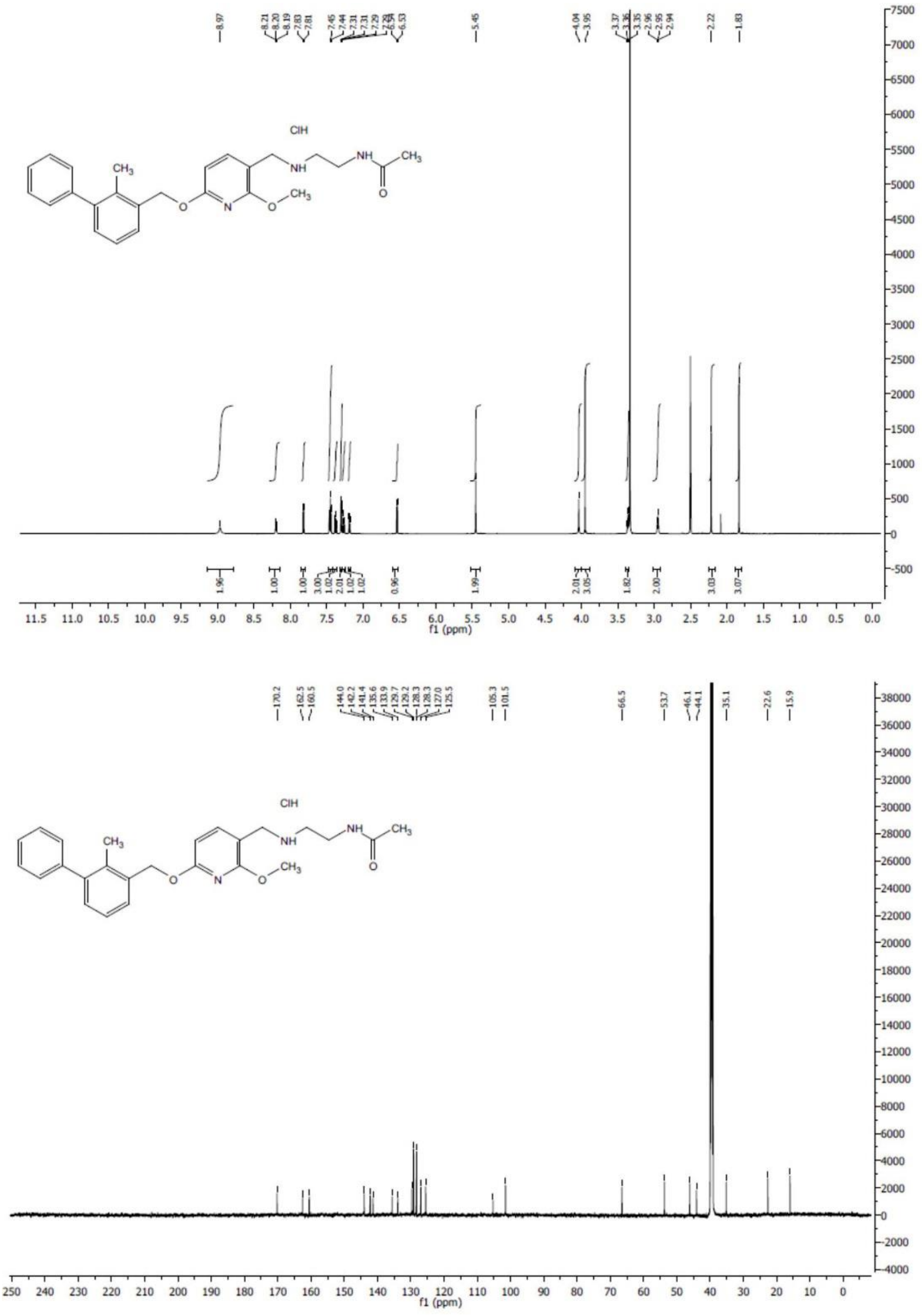

Figure S15

BMS-202 analysis. ^{1}H NMR (upper panel) and ^{13}C NMR (bottom panel).

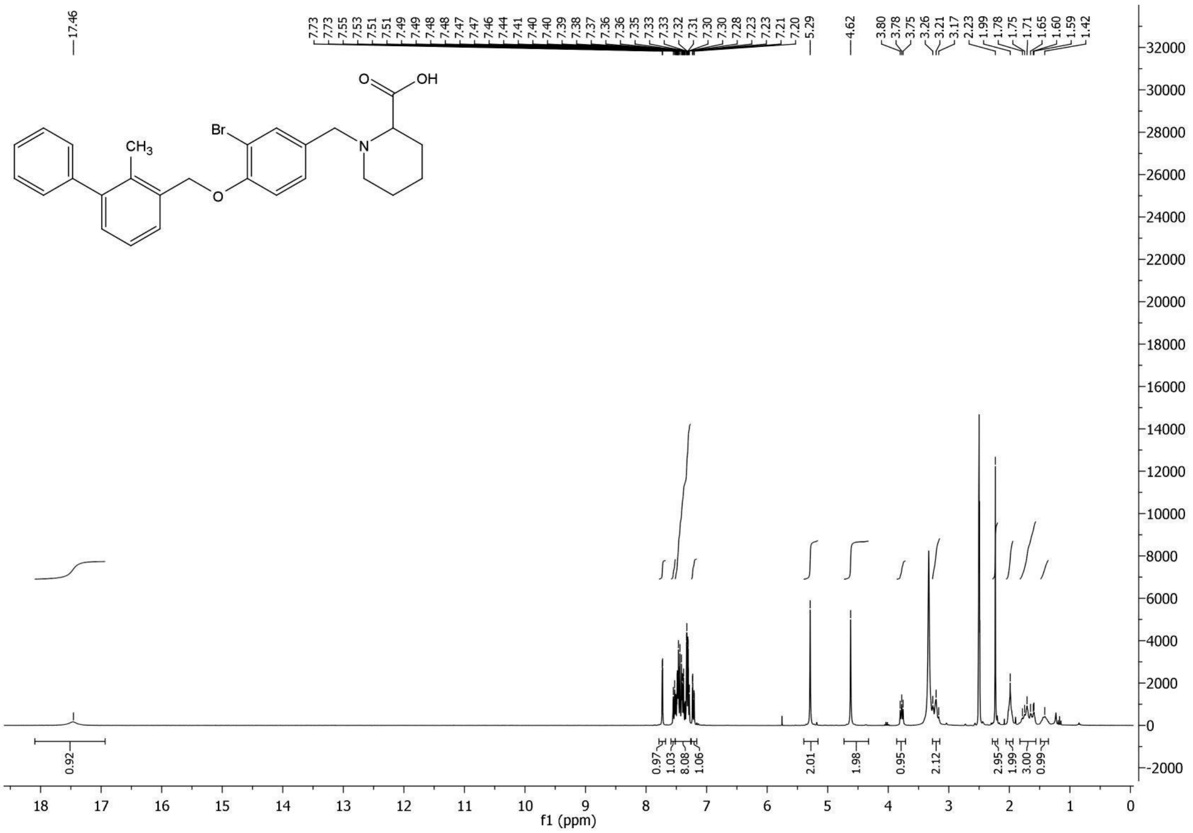

Figure S16

BMS-8 analysis. ^{1}H NMR.
